# Yeast α-arrestin Art2 is the key regulator of ubiquitylation-dependent endocytosis of plasma membrane vitamin B1 transporters

**DOI:** 10.1371/journal.pbio.3000512

**Published:** 2019-10-28

**Authors:** Jérôme Savocco, Sylvain Nootens, Wilhelmine Afokpa, Mathilde Bausart, Xiaoqian Chen, Jennifer Villers, Henri-François Renard, Martine Prévost, Ruddy Wattiez, Pierre Morsomme

**Affiliations:** 1 Louvain Institute of Biomolecular Science and Technology, Université catholique de Louvain, Louvain-la-Neuve, Belgium; 2 Structure and Function of Biological Membranes, Université libre de Bruxelles, Brussels, Belgium; 3 Department of Proteomics and Microbiology, Research Institute for Biosciences, Université de Mons, Mons, Belgium; UT Southwestern Medical Center, UNITED STATES

## Abstract

Endocytosis of membrane proteins in yeast requires α-arrestin-mediated ubiquitylation by the ubiquitin ligase Rsp5. Yet, the diversity of α-arrestin targets studied is restricted to a small subset of plasma membrane (PM) proteins. Here, we performed quantitative proteomics to identify new targets of 12 α-arrestins and gained insight into the diversity of pathways affected by α-arrestins, including the cell wall integrity pathway and PM–endoplasmic reticulum contact sites. We found that Art2 is the main regulator of substrate- and stress-induced ubiquitylation and endocytosis of the thiamine (vitamin B_1_) transporters: Thi7, nicotinamide riboside transporter 1 (Nrt1), and Thi72. Genetic screening allowed for the isolation of transport-defective Thi7 mutants, which impaired thiamine-induced endocytosis. Coexpression of inactive mutants with wild-type Thi7 revealed that both transporter conformation and transport activity are important to induce endocytosis. Finally, we provide evidence that Art2 mediated Thi7 endocytosis is regulated by the target of rapamycin complex 1 (TORC1) and requires the Sit4 phosphatase but is not inhibited by the Npr1 kinase.

## Abstract 2

A combination of proteomics, protein modeling, and molecular biology sheds light on how endocytosis of the plasma membrane vitamin B1 transporter Thi7 in yeast is regulated by the α-arrestin Art2.

## Introduction

In order to adapt to environmental cues, cells must constantly regulate the protein and lipid composition of their plasma membrane (PM); one such mechanism to ensure this is endocytosis. The endocytosis of many yeast transporters is triggered by the addition of an excess of their substrate or ligand [1–3]. In yeast, this process is triggered by protein ubiquitylation, catalyzed by a single ubiquitin ligase Rsp5. Similar to the human ortholog Nedd4, Rsp5 is a family member of the homologous to E6 associated protein carboxyl terminus (HECT)-type ubiquitin ligases. The common feature between all Rsp5 targets is the presence of an [L/V/P]PxY motif, which interacts with the WW domains of Rsp5 [4].

The yeast genome encodes approximately 240 PM proteins (UniProtKB, *Saccharomyces* Genome Database). Many PM proteins do not have [L/V/P]PxY motifs, yet they can still be endocytosed in an Rsp5-dependent manner [3,5–7]. In this case, the action of Rsp5 is mediated by [L/V/P]PxY motif–containing adaptors referred to as α-arrestins, which bind to both membrane proteins and Rsp5 [6,8]. In yeast, there are 13 α-arrestins, including the arrestin-related trafficking adaptors Art1 to 10 and the fungal-specific variants Bul1 to 3 [7,9,10]. The current model proposes that α-arrestins interact with Rsp5 via their [L/V/P]PxY motifs following an endocytic stimulus, and the complex translocates to the site of ubiquitylation, where the α-arrestin shuttles Rsp5 within close proximity to its target [6,7,11–14]. The function and specificity of α-arrestins are modulated by posttranslational modifications such as ubiquitylation and phosphorylation. α-arrestins are ubiquitylated by Rsp5, a modification that appears to be required for translocation of the Rsp5-adaptor complex to the PM [6]. However, the effect of α-arrestin ubiquitylation on target interaction remains unclear. For example, Art9 ubiquitylation is required for its interaction with Vps23 [12,15], whereas Art8 does not need ubiquitylation to bind to hexose transporter 6 (Hxt6) [16]. In addition, polyubiquitylation of specific lysine residues targets α-arrestins for proteasomal degradation [16,17]. It is generally assumed that phosphorylation of α-arrestins impairs their ability to mediate endocytosis [18]. Indeed, phosphorylated α-arrestins interact with 14-3-3 proteins (Bmh1/2) and are no longer able to mediate target ubiquitylation [13,19,20]. The kinases and phosphatases involved in this phospho-regulation depend on endocytosis-triggering stimulus. The antagonistic effects of the Npr1 kinase and the Sit4 phosphatase regulate α-arrestin- (Art1 and Bul1/2) mediated endocytosis of amino acid transporters, such as Gap1 and Can1, and the Bul1-mediated endocytosis of the lactate transporter Jen1 [13,19,21]. Similarly, the Snf1 kinase and the Glc7/Reg1 phosphatase are involved in the regulation of Art4 and Art7, thereby mediating endocytosis of carbon source transporters [14,20]. Other kinases, such as Ypk1 and PKA kinases, appear to phosphorylate Art4, whereas the casein kinases Yck1/2 phosphorylate Art9 [22,23]. The dephosphorylation of Art6 and Art4/7 by the calcineurin phosphatase mediates endocytosis of the dicarboxylic amino acid transporter Dip5 and the α-factor receptor Ste2, respectively [23–25].

The molecular mechanism explaining the signaling pathway going from substrate sensing to α-arrestin-regulated endocytosis is not clear. Focus on the permeases Fur4 (uracil) and Can1 (arginine) suggests that PM transport activity triggers a structural rearrangement from an outward to inward conformation. This disrupts the interaction between the amino-terminal cytosolic tail and cytosolic loops, exposing an α-arrestin binding sequence and/or a degradation sequence (hereafter called a degron) localized at the cytosolic amino-terminal tail [26–29]. In addition, the metabolization of accumulated substrate activates the α-arrestins to interact with transporters and thus leads to their endocytosis. For instance, the accumulation of arginine appears to stimulate target of rapamycin complex 1 (TORC1) kinase, which simultaneously inhibits Npr1 kinase and stimulates Sit4 phosphatase, resulting in the Art1-mediated ubiquitylation of Can1 [27,29]. However in some cases (e.g., Gap1 and Jen1), ubiquitylation-induced endocytosis is initiated via the activation of the α-arrestin in response to substrate accumulation, and no additional structural rearrangement appears to be required [27,29,30]. Alternatively, a recent study demonstrated that the activation of TORC1 could be linked to the activity of the proton pump plasma membrane ATPase 1 (Pma1) and H^+^ uptake by H^+^-amino acids symporters [31]. Although the available models are increasingly complex, a global view of α-arrestin activation for the wide diversity of PM proteins is still lacking. To broaden our understanding of α-arrestin integration in cellular homeostasis, we propose to study a more diverse set of PM proteins.

Here, we developed a methodology to analyze the effect of each α-arrestin on the adaptation of the PM protein landscape using quantitative proteomics. This approach allowed us to identify new targets for 12 α-arrestins. Our data also provide insight into the diversity of pathways affected by α-arrestins, such as the cell wall integrity pathway or the PM–endoplasmic reticulum (ER) tethering system. Among the identified targets, we focused on the poorly characterized thiamine (vitamin B_1_) transporter Thi7. Thiamine is the precursor of thiamine pyrophosphate (TPP), a coenzyme involved in both carbohydrate and amino acid metabolism [32]. Therefore, Thi7 has a global effect on cellular metabolism. We describe a new role for the Art2 α-arrestin as a mediator of the substrate- and stress-induced ubiquitylation and endocytosis of the thiamine transporter Thi7. The action of Art2 extends to the endocytosis of Thi7 homologs, the nicotinamide riboside transporter 1 (Nrt1) and Thi72. We also investigated the underlying mechanism of Thi7 endocytosis in response to thiamine transport and describe that both transporter conformation and transport activity are essential to induce endocytosis. Our data suggest that thiamine- and stress-induced Art2-mediated Thi7 endocytosis requires the presence of the Sit4 phosphatase. Furthermore, we propose that thiamine-induced Thi7 down-regulation is regulated by the TORC1 complex although it is not inhibited by the Npr1 kinase.

## Results

### Cycloheximide treatment affects the abundance of 33 PM proteins

To identify new PM proteins that require α-arrestins for endocytosis, we performed a quantitative proteomic screening. We analyzed and compared the PM proteome for each *artΔ* strain to a wild-type (WT) strain after a 90-min cycloheximide (CHX) treatment. CHX is a translation inhibitor and can trigger the endocytosis of several transporters in a substrate-independent manner [6,7]. We searched for proteins that underwent CHX-induced endocytosis in a WT strain but that remained stabilized at the PM in an *artΔ* strain (Tables 1 and 2). We identified 93 different PM proteins (S1 Table), which account for approximately 39% of the total number of PM proteins reported in databases (245 in *Saccharomyces* Genome Database and 234 in UniProtKB). The first step of the analysis consisted in the comparison of protein abundance in a CHX-treated WT strain (WT+CHX) and a mock-treated WT strain (WT ctrl) in order to identify proteins affected by the treatment (Table 1 and S2 Table). We observed that 24 integral PM proteins, mostly transporters, were less abundant in the WT+CHX than in the WT ctrl (in blue on Table 1), suggesting that CHX stimulates their endocytosis. In comparison, 9 proteins were more abundant in the WT+CHX than in the WT ctrl (in red on Table 1), suggesting that these proteins accumulate at the PM in response to CHX.

**Table 1:** CHX treatment affects the abundance of 33 PM proteins. Fold-change ratios of the abundance of PM proteins identified in the proteomic screening after CHX treatment. The first two columns indicate the UniProtKB accession numbers and names of proteins. The third column corresponds to the WT+CHX/WT ctrl ratio, specifying the protein relative abundance in the CHX-treated WT divided by its relative abundance in the mock-treated WT. The same total amount of proteins was analyzed in both conditions. The blue and red colors specify whether the ratio is significantly lower or higher than one after hypothesis testing (i.e., with a *p*-value < 0.05), respectively. A protein with a blue ratio (WT+CHX/WT ctrl < 1) is less abundant in the WT+CHX than in the WT ctrl, indicating a decrease in abundance upon CHX treatment. Inversely, a protein displaying a red ratio (WT+CHX/WT ctrl > 1) is more abundant in the WT+CHX than in the WT ctrl, suggesting an increase of the protein abundance upon CHX treatment. Proteins are classified according to their fold change after CHX treatment of WT strain, from the greatest decrease (Ptr2) to the greatest increase in abundance (Zeo1). Displayed ratios correspond to the geometric means from 6 independent experiments.

Accession	Name	WT + CHX/WT ctrl
P32901	PTR2	0.03
Q05998	THI7	0.14
P38631	FKS1	0.18
P04817	CAN1	0.19
Q01896	ENA2	0.24
P32791	FRE1	0.31
P32466	HXT3	0.35
P38085	TAT1	0.38
Q06689	INA1	0.41
P32465	HXT1	0.42
P39004	HXT7	0.44
P32467	HXT4	0.48
P38079	YRO2	0.55
P40088	FTR1	0.55
P05030	PMA1	0.58
Q12256	TPO4	0.58
P38993	FET3	0.60
P49573	CTR1	0.63
P40474	QDR2	0.64
P22146	GAS1	0.74
P33302	PDR5	0.84
P32568	SNQ2	0.85
P53049	YOR1	0.87
P23292	YCK2	0.88
P48231	TCB2	1.50
P01120	RAS2	1.55
Q12466	TCB1	1.88
Q00245	RHO3	1.95
Q03640	TCB3	2.05
P38250	IST2	2.74
Q12207	NCE102	3.16
P06780	RHO1	3.81
Q08245	ZEO1	16.42

Abbreviations: CHX, cycloheximide; PM, plasma membrane; WT, wild-type

**Table 2:** Impact of α-arrestin deletion on the abundance of PM proteins affected by the CHX treatment. Fold-change ratios of the abundance of PM proteins identified in Table 1 between the CHX-treated *artΔ* and WT strains. The first two columns indicate the UniProtKB accession number and name of proteins. The remaining columns correspond to *artΔ*+CHX/WT ctrl ratio, specifying the protein relative abundance in the CHX-treated arrestin-knockout strains divided by its relative abundance in the CHX-treated WT. The same total amount of proteins was analyzed in all conditions tested. The red and blue colors specify whether the ratio is significantly higher or lower than 1 after hypothesis testing (i.e., with a *p*-value < 0.05), respectively. A protein displaying a red ratio (*artΔ*+CHX/WT+CHX > 1) is more abundant in the *artΔ*+CHX than in the WT+CHX condition. We interpret an increase in surface protein upon α-arrestin deletion (i.e., indicated in red) as reflecting a defect in internalization of that PM protein. Inversely, a protein with a blue ratio (*artΔ*+CHX/WT+CHX < 1) is less abundant in the *artΔ*+CHX than in the WT+CHX. White ratios are not significantly different from one upon hypothesis testing, regardless of their absolute values. Proteins are classified as in Table 1. Displayed ratios correspond to the geometric means from 3 independent experiments.

Accession	Name	*art1Δ+*CHX/WT+CHX	*art2Δ+*CHX/WT+CHX	*art3Δ+*CHX/WT+CHX	*art4Δ+*CHX/WT+CHX	*art5Δ+*CHX/WT+CHX	*art6Δ+*CHX/WT+CHX	*art7Δ*+CHX/WT+CHX	*art8Δ*+CHX/WT+CHX	*art9Δ*+CHX/WT+CHX	*art10Δ*+CHX/WT+CHX	*bul1Δ*+CHX/WT+CHX	*bul2Δ*+CHX/WT+CHX
P32901	PTR2	9.0	10.8	10.8	6.2	11.2	8.6	2.6	4.4	2.6	2.3	17.7	2.4
Q05998	THI7	1.2	4.1	1.0	0.6	0.8	0.4	0.3	0.3	11.3	0.3	0.5	0.3
P38631	FKS1	5.2	3.0	5.5	2.5	3.2	2.1	1.7	1.4	3.5	1.8	2.9	1.0
P04817	CAN1	3.5	2.0	1.4	1.4	1.2	1.4	1.5	1.7	6.5	1.2	1.2	1.8
Q01896	ENA2	1.7	2.4	6.8	4.7	1.5	1.1	1.5	1.4	1.7	1.4	0.5	1.1
P32791	FRE1	5.4	4.2	4.7	3.1	3.6	2.4	2.5	1.9	2.8	1.9	2.8	1.7
P32466	HXT3	3.0	1.5	2.1	8.4	1.3	0.7	1.7	0.8	3.6	1.2	1.5	0.7
P38085	TAT1	2.4	1.8	2.2	1.1	1.6	1.6	1.4	1.4	0.7	1.2	1.2	1.6
Q06689	INA1	1.2	0.9	1.6	0.8	0.8	0.4	0.8	0.4	0.5	0.7	0.1	0.3
P32465	HXT1	4.6	2.8	4.4	9.3	2.6	1.6	2.2	1.8	1.7	2.4	1.4	1.9
P39004	HXT7	0.6	0.4	0.3	1.1	0.6	0.2	0.9	0.6	0.1	0.7	0.1	0.4
P32467	HXT4	0.7	0.6	0.6	2.8	0.5	0.6	0.7	0.5	0.4	0.6	0.2	0.5
P38079	YRO2	3.9	1.2	2.8	1.2	1.5	0.6	2.0	1.1	0.4	1.5	1.7	0.5
P40088	FTR1	2.2	1.0	1.8	1.6	1.7	0.8	1.3	0.6	2.1	1.1	1.2	0.5
P05030	PMA1	4.8	0.7	2.9	1.6	2.2	0.5	1.3	0.4	0.4	0.8	0.6	0.3
Q12256	TPO4	3.2	1.5	2.1	2.1	1.8	1.4	1.6	1.0	0.2	1.1	0.8	1.0
P38993	FET3	2.8	0.8	1.4	1.7	1.8	1.1	1.2	0.6	2.1	0.9	1.1	0.7
P49573	CTR1	1.2	1.3	2.0	1.5	1.3	1.1	1.4	0.9	0.4	1.1	1.1	0.8
P40474	QDR2	0.7	0.7	1.0	0.7	0.6	0.4	1.0	0.7	1.2	0.9	0.3	0.4
P22146	GAS1	1.7	1.2	2.0	1.0	1.1	0.3	1.4	0.7	0.2	1.1	0.7	0.3
P33302	PDR5	0.8	2.4	4.7	2.9	2.4	1.1	3.0	1.4	2.1	2.2	0.5	1.4
P32568	SNQ2	1.9	1.5	2.6	1.3	1.3	0.8	2.4	1.1	2.6	1.5	1.4	0.7
P53049	YOR1	2.3	1.5	1.8	1.5	1.7	0.8	1.5	1.1	1.4	1.1	1.1	0.7
P23292	YCK2	1.4	0.8	1.3	1.2	1.1	0.7	1.1	0.8	0.9	0.9	0.8	0.7
P48231	TCB2	0.4	0.8	0.7	0.8	0.5	0.5	0.7	0.7	1.1	0.8	0.7	0.5
P01120	RAS2	1.1	0.8	1.3	1.0	1.1	0.7	1.0	0.6	0.1	0.9	0.7	0.5
Q12466	TCB1	0.4	0.4	0.5	0.7	0.5	0.3	0.7	0.6	0.6	0.7	0.4	0.3
Q00245	RHO3	1.0	0.9	1.0	1.0	1.0	0.5	0.9	0.7	1.1	0.7	0.4	0.5
Q03640	TCB3	0.4	0.5	0.7	0.7	0.5	0.3	0.6	0.5	0.8	0.6	0.4	0.3
P38250	IST2	0.5	0.4	0.6	0.8	0.6	0.5	0.6	0.5	1.1	0.9	0.6	0.3
Q12207	NCE102	0.8	0.5	0.8	0.4	0.6	0.4	0.8	0.7	0.3	0.8	0.5	0.4
P06780	RHO1	0.7	0.6	0.8	0.6	0.8	0.5	0.8	0.7	0.5	0.7	0.8	0.4
Q08245	ZEO1	0.6	0.9	1.3	0.4	0.9	0.4	1.1	0.9	0.2	0.9	0.5	0.4

Abbreviations: CHX, cycloheximide; PM, plasma membrane; WT, wild-type

To determine whether CHX-induced endocytosis is α-arrestin dependent, we compared the effect of α-arrestin deletion (*artΔ*+CHX vs WT+CHX) on PM protein abundance of the same 24 proteins that were depleted upon CHX treatment (Table 2 and S2 Table). We interpreted an increase in surface protein upon α-arrestin deletion (indicated in red) as reflecting a defect in internalization of that PM protein. It appeared that the endocytosis of some proteins was affected by only one α-arrestin, such as peptide transporter 2 (Ptr2). For example, in the absence of Bul1 (*bul1Δ)*, CHX treatment failed to reduce the abundance of Ptr2 (Table 2). In contrast, the abundance of other proteins was affected by several α-arrestins. For example, the catalytic subunit of the β-1,3-D-glucan synthase Fks1 appears to be stabilized in several *artΔ* strains. These data suggest that several α-arrestins are involved in the regulation of Fks1 abundance at the PM in a nonredundant manner, since the lack of each one independently results in an increased Fks1 abundance. As expected, our data are consistent with previously described interactions between model transporters and α-arrestins, such as the Bul1 dependency of Ptr2 endocytosis after CHX treatment [33], the Art1 dependency of Can1 endocytosis after substrate and CHX treatment [6], and the regulation of Hxt1 and Hxt3 by Art4 and Art7 after 2-deoxyglucose treatment [14]. However, our data did not show some of the previously described effects of α-arrestins on some transporters. For example, the role of Art8 in Hxt7 endocytosis upon glucose depletion [16] was not observed in our current working condition, most likely because cells were grown in glucose-containing medium.

Unexpectedly, proteins such as Hxt4, Hxt7, Pma1, or Gas1 were less abundant at the PM in some *artΔ* strains after CHX treatment (Table 2). This could be due to increased protein degradation in the absence of the α-arrestin, the involvement of a stimulated compensatory α-arrestin-independent endocytic mechanism, or reduced gene expression as recently illustrated for Hxt6 and Hxt7 in an *art8Δ* strain [16]. Of note, the glycosylphosphatidylinositol (GPI)-anchored protein Gas1 does not have any cytosolic part and is therefore unable to interact directly with α-arrestins. Yet, its abundance is decreased in the *art9Δ* and *bul2Δ* backgrounds compared to the WT condition. To our knowledge, this result is the first example of an effect of α-arrestin on the abundance of GPI-anchored proteins.

Among the nine proteins that were more abundant after the CHX treatment than in the control condition, we primarily identified peripheral proteins or proteins tightly associated with the PM. Indeed, the eisosome component Nce102 was the only identified intrinsic PM protein. These nine proteins are involved in either the regulation of PM lipid composition (as the ER–PM tether components Tcb1/3 and Ist2), PM organization (Nce102), cell integrity pathway (Rho1, Zeo1), or polarized secretion (Rho3). Moreover, we identified the guanosine triphosphate (GTP)-binding protein Ras2, previously shown to physically interact with Art1, Art4, and Art7 [34] and with two other identified proteins of our screening, the ATP-binding cassette (ABC) transporters Yor1 and Pdr5 [35]. In our proteomic analysis, Ras2 abundance is decreased in *art9Δ* and *bul2Δ* conditions.

### CHX- and thiamine-induced endocytosis of Thi7 is impaired in the art2Δ strain

The abundance of the high-affinity thiamine transporter Thi7 was highly affected by CHX treatment. In the CHX-treated WT, Thi7 abundance is reduced to 14% of its value after mock treatment. Moreover, following CHX treatment, Thi7 was 4 and 11 times more abundant at the PM of *art2Δ* and *art9Δ* strains than of the WT, suggesting that its CHX-induced endocytosis is mediated by Art2 and Art9. These results indicate that these two α-arrestins are not fully redundant, as the presence of *ART9* in *art2Δ* does not rescue the down-regulation of Thi7 upon CHX treatment and vice versa.

To assess protein trafficking, we engineered N- and C-terminal green fluorescent protein (GFP) fusions of Thi7 expressed under the THI7 promoter and observed protein localization following CHX treatment. The same was carried out for the two low-affinity thiamine transporter homologs Nrt1 and Thi72 (84% and 87% identity with Thi7, respectively). Phenotypic growth tests confirmed the activity of C-terminal GFP fusions (S1 Fig). The growth defect triggered by a toxic analog of thiamine, oxythiamine, was used as readout of transporter activity. The *thi7Δnrt1Δthi72Δ* strain, which is defective for thiamine transport and expresses native N- and C-terminal GFP proteins, was grown on thiamine-free oxythiamine-supplemented medium. As expected, the *thi7Δnrt1Δthi72Δ* strain transformed with an empty plasmid was resistant to oxythiamine. We observed that *THI7*- and *THI72*-expressing strains were sensitive to oxythiamine, whereas the *NRT1*-expressing strains were less sensitive. The C-terminal GFP fusion proteins displayed the same phenotype as the corresponding native protein. Because thiamine is the physiological substrate of Thi7, thiamine uptake by the fusion proteins was also probed by testing growth on low thiamine concentration. The *thi7Δnrt1Δthi72Δthi4Δ* strain, which is deficient for thiamine transport and synthesis and expresses GFP-fused transporters, was grown on medium containing 1 nM to 100 μM thiamine. As expected, the *thi7Δnrt1Δthi72Δthi4Δ* strain that was transformed with the empty plasmid only grew in the presence of the highest concentration of thiamine because of unspecific thiamine entry (S1 Fig). In contrast, strains that expressed a transporter grew on all media regardless of thiamine concentration. Growth level of strains expressing the C-terminal GFP fusion proteins was similar to those expressing native transporters.

**Fig 1:**
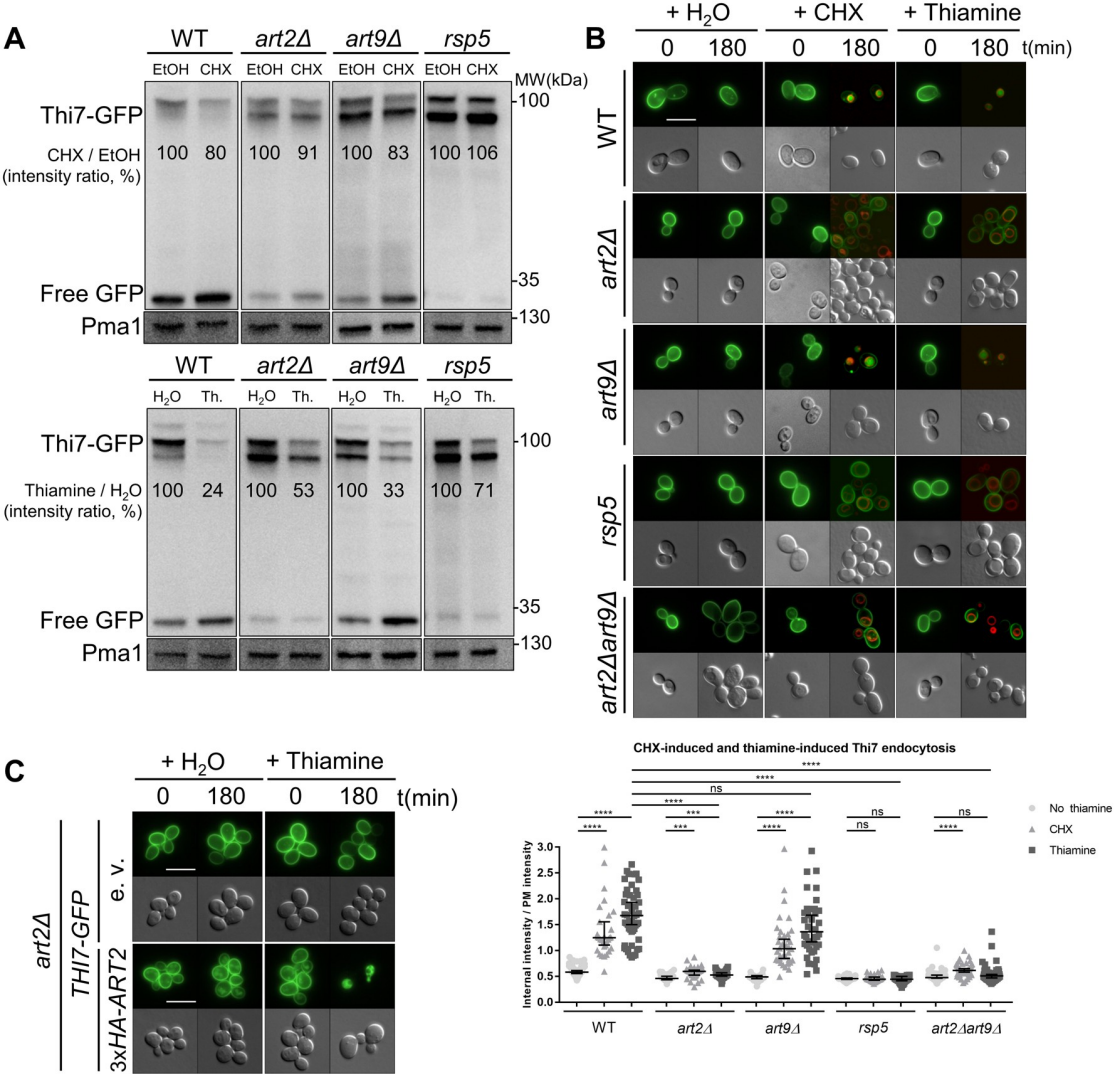
Art2 and Rsp5 are required for CHX- and thiamine-induced Thi7 endocytosis. (A) The WT, *art2Δ*, *art9Δ*, and *rsp5* strains expressing *THI7-GFP* under their endogenous promoter were grown in thiamine-free selective medium up to early-log phase and harvested after 120 min with CHX (final concentration; 50 μg/ml) or EtOH (top panel) and with thiamine (“Th.,” final concentration: 100 μM) or water (H_2_O) (bottom panel). Total protein extracts were immunoblotted with anti-GFP and anti-Pma1 as a loading control. Free GFP is the results of the degradation of Thi7-GFP in the vacuole. Thi7-GFP band intensity is normalized by the intensity of the Pma1-corresponding band. Representative of 3 experiments. (B) Localization of Thi7-GFP in the WT, *art2Δ*, *art9Δ*, *rsp5*, and *art2Δart9Δ* strains after addition of thiamine or CHX or mock-treated with water. The vacuolar membrane is stained with FM4–64. Quantification shows the ratio of internal-over-PM fluorescence intensity as described in Materials and methods section (*n* > 30 cells) (****p < 0.0001; ***p < 0.001; **p < 0.01; *p < 0.05). (C) Expression of *3xHA-ART2* restores thiamine-induced endocytosis of Thi7-GFP in the *art2Δ* background. *THI7-GFP* and either *3xHA-ART2* or its corresponding e.v. are expressed in the *art2Δ* strain, grown in thiamine-free selective medium, and complemented with thiamine (final concentration: 100 μM). Scale bar represents 5 μm. The numerical data are included in S1 Data. CHX, cycloheximide; EtOH, ethanol; e.v., empty vector; GFP, green fluorescent protein; ns, nonsignificant; PM, plasma membrane; Pma1, plasma membrane ATPase 1; WT, wild type.

To confirm that the high abundance of Thi7 following CHX treatment in *art2Δ* and *art9Δ* strains resulted from the impairment of Thi7 endocytosis, we analyzed Thi7-GFP fluorescence in WT and the different *artΔ* strains. In addition, we included the analysis of Thi7-GFP endocytosis in the mutant strain *rsp5*, which is deficient for *RSP5* expression [5,36]. Strains were grown in thiamine-free medium to allow *THI7-GFP* expression and localization at the PM before mock- or CHX treatment [37]. Specific immunoblot signal for Thi7-GFP appears as two major bands in the 100-kDa range (Fig 1A). Moreover, free GFP, which is more resistant to vacuolar degradation, is detected at about 27 kDa, suggesting that our constructs can be targeted to the vacuole. CHX treatment decreased Thi7-GFP abundance in the WT strain compared to the mock-treated condition. Reduced Thi7-GFP abundance was attenuated in *art2Δ* and *rsp5* backgrounds but not in *art9Δ* (Fig 1A). Free GFP signal is less intense in *art2Δ* and almost absent in *rsp5* strains. Of note, the measured endocytosis rates of Thi7 are lower in immunoblot experiments than in the proteomic screening because of two major differences in the methodology. On the one hand, mass spectrometry was performed on PM-enriched fractions to focus on the abundance of Thi7 at the PM, whereas immunoblots were performed on total protein extracts. In total extracts, the whole pool of Thi7 is identified (including Thi7 en route to the PM or the vacuole), lessening the ratio of Thi7 degradation. On the other hand, the protein extracts used for immunoblots were performed on cells growing in thiamine-free synthetic medium, maximizing *THI7* expression, whereas mass spectrometry was performed in YD medium (containing thiamine), in which *THI7* expression is low. This may affect the relative proportion of endocytosed Thi7. In parallel, we assessed Thi7-GFP localization after CHX treatment and observed that the construct was blocked at the PM in *art2Δ* and *rsp5* backgrounds (Fig 1B). By contrast, Thi7-GFP localized to the vacuolar compartment in WT and *art9Δ* strains (Fig 1B). The *art2Δart9Δ* strain has the same phenotype as the *art2Δ* strain (Fig 1B). Together, these results demonstrate that CHX-induced endocytosis/degradation of Thi7 is Rsp5 and Art2 dependent.

To assess trafficking at a condition that was more physiologically relevant, we measured localization of Thi7-GFP following thiamine treatment of cells precultured in thiamine-free medium (Fig 1A). Thiamine is the natural substrate of Thi7, and when added in excess, it inhibits *THI7* gene expression [37]. In WT and *art9Δ* strains, thiamine treatment decreased Thi7-GFP protein level by 67%–76%, indicating that these transporters were degraded. By contrast, thiamine treatment of *art2Δ* and *rsp5* strains partially inhibited Thi7-GFP endocytosis. Thi7-GFP protein levels were reduced by 47% and 29% in *art2Δ* and *rsp5* strains, respectively Consistent with these observations, free GFP level was reduced in *art2Δ* and *rsp5* compared to WT and *art9Δ* strains. We also monitored the localization of Thi7-GFP after thiamine treatment in WT, *art2Δ*, *art9Δ*, *art2Δart9Δ*, and *rsp5* strains by microscopy. GFP signal appeared primarily targeted to the vacuole after 3-h thiamine treatment of WT and *art9Δ* strain (Fig 1B) as in the remaining *artΔ* strains (S2 Fig). However, this is not observed in *art2Δ*, *art2Δart9Δ*, and *rsp5* strains, and the GFP signal appeared to be stabilized at the PM. Finally, expression of *3xHA-ART2* in *art2Δ* fully rescued thiamine-induced Thi7 endocytosis (Fig 1C). These results demonstrate that thiamine-induced endocytosis/degradation of Thi7 is Rsp5 and Art2 dependent.

### The deletion of ART2 and ART9 impairs endocytosis of the two putative low-affinity thiamine transporters Nrt1 and Thi72

**Fig 2:**
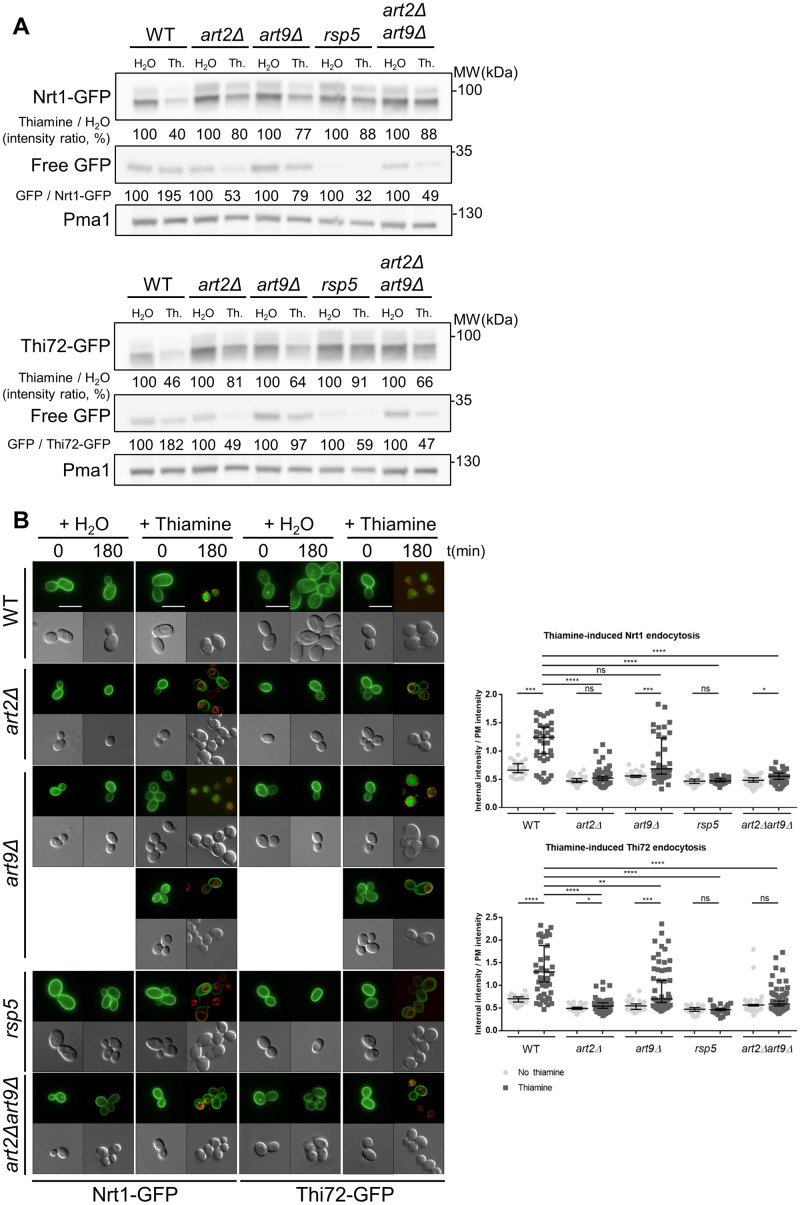
The endocytosis of the low-affinity thiamine transporters Nrt1 and Thi72 is also mediated by Art2. (A) Strains expressing *NRT1-GFP* (top panel) and *THI72-GFP* (bottom panel) under *THI7* endogenous promoter were grown in thiamine-free medium up to early-log phase and harvested after 120-min incubation with thiamine (“Th.”) at a concentration of 100 μM for total protein extractions. Extracts were immunoblotted with anti-GFP and anti-Pma1 as a loading control. Values are quantification of band intensity. (B) Localization of Nrt1-GFP or Thi72-GFP in WT, *art2Δ*, *art9Δ*, *rsp5*, and *art2Δart9Δ* strains after addition of an excess of thiamine into culture grown in thiamine-free medium. The vacuolar membrane is stained with FM4–64. Scale bar represents 5 μm. Quantification shows the ratio of internal-over-PM fluorescence intensity as described in Materials and methods section (*n* > 50 cells). (****p < 0.0001; ***p < 0.001; **p < 0.01; *p < 0.05) The numerical data are included in S2 Data. GFP, green fluorescent protein; Nrt1, nicotinamide riboside transporter 1; ns, nonsignificant; PM, plasma membrane; Pma1, plasma membrane ATPase 1; WT, wild type.

We extended our study to examine the effect of α-arrestin on endocytosis of low-affinity thiamine transporters. We examined their endocytosis after thiamine treatment (Fig 2). Thiamine reduced Nrt1-GFP and Thi72-GFP protein abundance by 60% in the WT strain. This reduction was partially inhibited in the *art2Δ*, *art9Δ*, *rsp5*, and *art2Δart9Δ* strains (Fig 2A). Of note, the effect of *ART9* deletion on Nrt1-GFP and Thi72-GFP endocytosis is different from the one observed on Thi7-GFP (Fig 1A). We also calculated the ratio of free GFP normalized to Nrt1-GFP or Thi72-GFP abundance in the different strains to estimate the proportion of transporters targeted to vacuolar degradation. The free GFP abundance is 2-fold higher in thiamine-treated than in the mock-treated WT for both transporters. In contrast, free GFP level is 2-fold lower in *art2Δ* after thiamine treatment than after mock-treatment, suggesting that degradation is strongly impaired. Although the effect observed in the *art9Δ* strain is less important than with *art2Δ*, it nevertheless suggests a lower degradation in *art9Δ* compared to the WT. Nrt1-GFP and Thi72-GFP, which under thiamine-free conditions are localized to the PM, were targeted to the vacuole in the WT strain treated with thiamine (Fig 2B). The deletion of *ART2* reduced the extent of Nrt1 and Thi72 degradation by stabilizing the transporters at the PM. In the *art9Δ* strain, we observed an intermediate phenotype: GFP signal appeared to localize within the PM of approximately 50% of cells, whereas it could be clearly observed in the vacuoles of the remaining cells. Of note, *ART9* deletion had no previous effect on the Thi7 transporter. Finally, we observed Nrt1-GFP and Thi72-GFP localization after thiamine treatment in all the other *artΔ* strains, and no difference was observed with WT strain (S2 Fig). Taken together, these results emphasize a general role of Art2 in the endocytosis of the three thiamine transporters, whereas Art9 function seems to be restricted to the endocytosis of the low-affinity homologs, Nrt1 and Thi72.

### Thi7 is ubiquitylated within the cytosolic C terminus in an Art2-dependent manner

**Fig 3:**
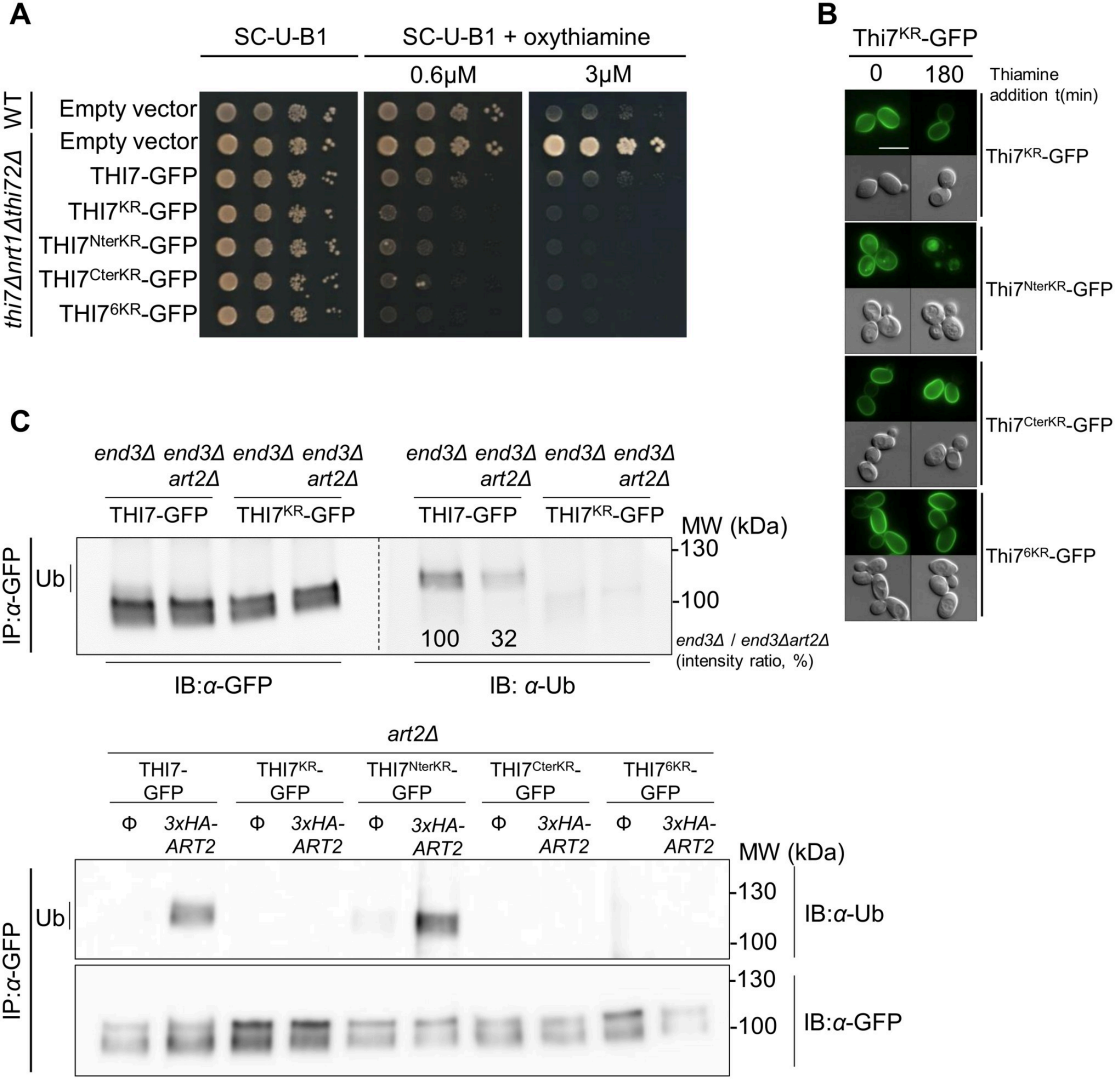
The Art2-dependent ubiquitylation of Thi7-GFP C terminus is required for its endocytosis. (A) GFP-tagged KR-mutated THI7 was expressed in a *thi7Δnrt1Δthi72Δ* strain and grown in a thiamine-free medium. Serial 10-fold dilutions of yeast cells in early log phase were spotted on thiamine-free oxythiamine-containing medium. The expression of pRS416 (empty vector) and the nontagged version were used as controls. Oxythiamine was transported by all the KR Thi7 mutants (B) Localization of the different KR mutant versions of Thi7-GFP in the WT strain after thiamine treatment (final concentration: 100 μM): Thi7^KR^-GFP, Thi7^NterKR^-GFP, Thi7^CterKR^-GFP, and Thi7^Cter6KR^-GFP. (C) Top: *THI7-GFP* and *THI7*^*KR*^*-GFP* were expressed in *end3Δ* and *end3Δart2Δ* strains and grown in thiamine-free medium to early log phase before a 30-min incubation with 100 μM thiamine. Bottom: *THI7-GFP*, Thi7^KR^-GFP, Thi7^NterKR^-GFP, Thi7^CterKR^-GFP, or Thi7^Cter6KR^-GFP were expressed in *art2Δ* strain (complemented or not with *3xHA-ART2)* and grown in thiamine-free medium to early log phase before a 30-min incubation with 100 μM thiamine. After solubilization, IP of transporters was performed using the GFP-Trap kit. Samples were immunoblotted with anti-GFP and anti-Ub. Quantification is based on the intensity of the Ub band and normalized to the intensity of the corresponding GFP signal. GFP, green fluorescent protein; IB, immunoblotting; IP, immunoprecipitation; KR, lysine-to-arginine; Ub, ubiquitin; WT, wild type.

We designed various lysine-to-arginine (KR) mutants of Thi7-GFP to identify which lysine residues are essential for Rsp5-dependent ubiquitylation and endocytosis (Fig 3). We performed phenotypic growth tests on oxythiamine-containing medium of the *thi7Δnrt1Δthi72Δ* strain expressing the KR mutants to ensure that mutations do not impair transport activity (Fig 3A). Thiamine treatment had no effect on the endocytosis of the Thi7^KR^-GFP mutant, in which all cytosolic N- and C-terminal lysine residues were mutated to arginine (Fig 3B). Similarly, endocytosis is completely abrogated upon mutation of C-terminal lysine residues (Thi7^CterKR^-GFP mutant), suggesting ubiquitylation takes place at the C terminus. Indeed, endocytosis occurred if lysine residue mutations were restricted to the cytosolic N-terminal tail (Thi7^NterKR^-GFP mutant). To confine our search to 6 C-terminal lysine residues, which were predicted to be ubiquitylated [38], we engineered the Thi7^6KR^-GFP mutant. Similar to the Thi7^KR^-GFP and Thi7^CterKR^-GFP mutants, the Thi7^6KR^-GFP mutant is not endocytosed after thiamine treatment, suggesting that at least one of these lysine residues is essential for Thi7 endocytosis (Fig 3B).

To examine the role of Art2 in Thi7 ubiquitylation, we immunoprecipitated Thi7-GFP and Thi7^KR^-GFP from *end3Δ* and *end3Δart2Δ* backgrounds, in which endocytosis is impaired and ubiquitylated proteins remain stabilized within the PM. Using an anti-ubiquitin antibody, we detected a higher molecular weight signal than Thi7-GFP in the *end3Δ* strain, corresponding to a ubiquitylated form of the construct (Fig 3C, upper panel). The intensity of this signal was reduced (68%) in the *end3Δart2Δ* background, suggesting that Art2 is involved in the ubiquitylation of Thi7-GFP. However, the residual signal indicates that some ubiquitylation is Art2-independent. The absence of a ubiquitylated signal within the Thi7^KR^-GFP fusion protein, regardless of the background (Fig 3C, upper panel), supports the specificity of our model. Additionally, we tried to rescue the ubiquitylation process by expressing *3xHA-ART2* and the different constructs in *art2Δ* (Fig 3C, lower panel). In the absence of Art2, Thi7-GFP is not ubiquitylated. Whereas Thi7^KR^-GFP, Thi7^CterKR^-GFP, and Thi7^6KR^-GFP remained nonubiquitylated in both conditions, expression of *3xHA-ART2* in *art2Δ* restored ubiquitylation of Thi7-GFP and Thi7^NterKR^-GFP. Therefore, our data demonstrate that Thi7 is ubiquitylated in its cytosolic C-terminal tail and that its ubiquitylation is mainly Art2 dependent.

### An oxythiamine-based screening revealed specific transport-defective Thi7 mutants

**Fig 4:**
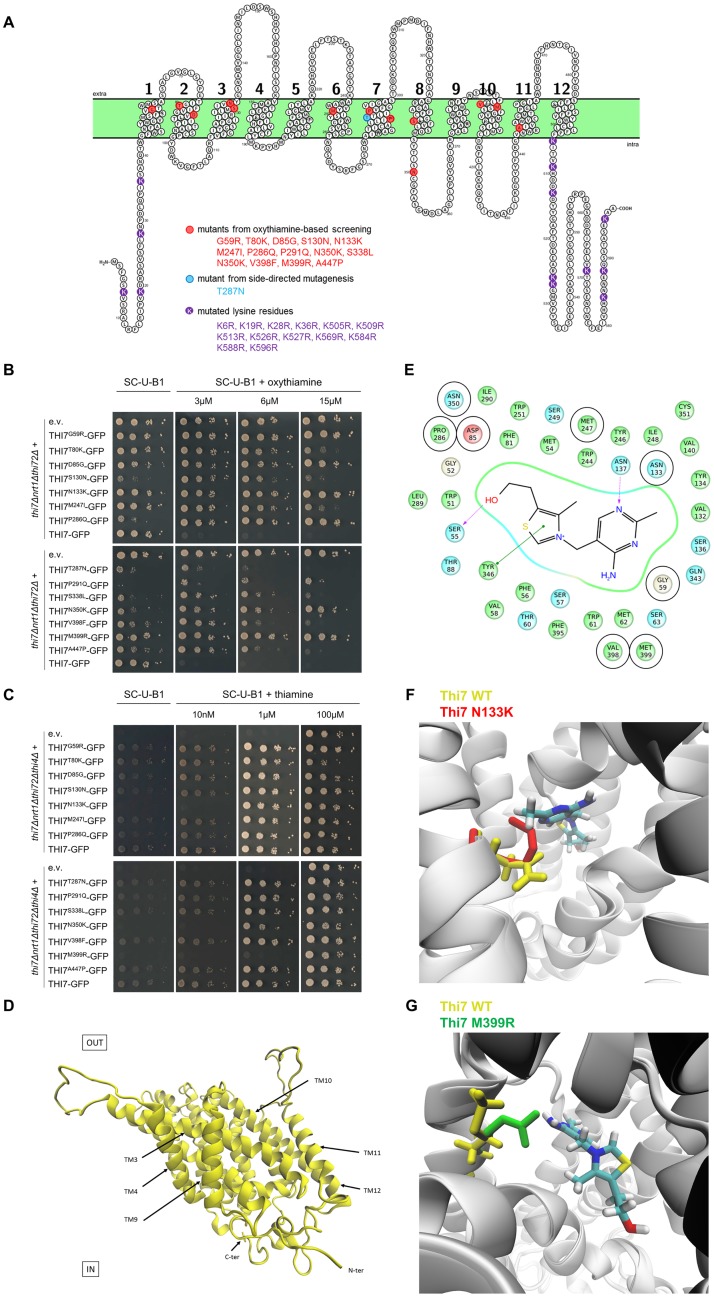
Selection of 14 single-point Thi7 mutants with reduced transport activity and structural modelling of Thi7. (A) Scheme of Thi7 topology generated by Protter software [43], representing the 14 single-point Thi7 mutants isolated by oxythiamine-based screening (red) or by side-directed mutagenesis (blue). The lysine residues mutated to generate Thi7^KR^ mutants are shown in purple. (B) Phenotypic growth test of *thi7Δnrt1Δthi72Δ* strain expressing single-point *THI7-GFP* mutants, WT *THI7-GFP*, or an e.v. on thiamine-free selective medium (SC-U-B1) supplemented or not with oxythiamine (final concentrations; 3, 6, and 15 μM). Representative of 5 experiments. (C) Phenotypic growth test of *thi7Δnrt1Δthi72Δthi4Δ* strain expressing single-point *THI7-GFP* mutants, WT *THI7-GFP*, or an e.v. on thiamine-free selective medium (SC-U-B1) or supplemented with thiamine (final concentrations; 10 nM, 1 μM, and 100 μM). Representative of 4 experiments. (D) A 3D model of Thi7 in an occluded state. (E) Proposed thiamine binding site in Thi7. Residues predicted to be at a distance equal to or less than 6 Å from the thiamine molecule are shown. Docking experiments predict that thiamine is stabilized by two hydrogen bridges with S55 and N137 (in purple) and one π-π interaction with Y346 (in green). Eight Thi7 mutants obtained in the oxythiamine-based screening result from mutation of specific residues surrounding the binding site (encircled in black: G59, D85, N133, M247, P286, N350, V398, M399). (F) Superimposition of the 3D model of WT Thi7 (residue N133 in yellow) in a thiamine-bound occluded state with the 3D model of Thi7^N133K^ (residue K133 in red) in an occluded state displays how the N133K mutation could affect the binding site and the stabilization of thiamine. (G) Superimposition of the 3D model of WT Thi7 (residue M399 in yellow) in a thiamine-bound occluded state with the 3D model of Thi7^M399R^ (residue R399 in green) in an occluded state displays how the M399R mutation could disturb the binding site and stabilization of thiamine. 3D, three-dimensional; C-ter, C terminus; e.v., empty vector; GFP, green fluorescent protein; KR, lysine-to-arginine; N-ter, N terminus; TM, transmembrane span; WT, wild type.

We focused next on the underlying mechanism of Thi7 endocytosis in response to thiamine transport. First, we found that Thi7-GFP is endocytosed after oxythiamine treatment of WT cells (S3 Fig), suggesting that substrate transport triggers Thi7-GFP endocytosis regardless of TPP-dependent enzyme activity. To address the underlying mechanism of Thi7 endocytosis in response to thiamine transport, an oxythiamine-based screening was completed in an attempt to isolate transport-defective Thi7 mutants. Thirteen single-point mutants were identified in this screen: Thi7^G59R^, Thi7^T80K^, Thi7^D85G^, Thi7^S130N^, Thi7^N133K^, Thi7^M247I^, Thi7^P286Q^, Thi7^P291Q^, Thi7^S338L^, Thi7^N350K^, Thi7^V398F^, Thi7^M399R^, and Thi7^A447P^. In addition, we generated a Thi7^T287N^ mutant that in a previous study reduced thiamine uptake activity by 75%–80% [39]. All but one mutation appears to be localized within transmembrane segments (Fig 4A). The *thi7Δnrt1Δthi72Δ* strain was retransformed with each isolated Thi7 mutant, and phenotypic growth tests confirmed that their expression induced resistance to varying extents to oxythiamine (Fig 4B). The strains expressing Thi7^G59R^, Thi7^D85G^, Thi7^N133K^, Thi7^P286Q^, Thi7^N350K^, or Thi7^M399R^ mutants appeared to be as resistant as the *thi7Δnrt1Δthi72Δ* strain up to 15 μM oxythiamine, suggesting a loss of function. At this concentration, growth defects are observed when *thi7Δnrt1Δthi72Δ* cells express Thi7^T80K^, Thi7^S130N^, Thi7^M247I^, Thi7^T287N^, Thi7^P291Q^, Thi7^S338L^, Thi7^V398F^, and Thi7^A447P^, indicating an intermediate functionality. Small variations of abundance between the mutants and WT Thi7 proteins were observed, but they were not correlated to resistance, confirming that the phenotypes resulted from partial functionality rather than impaired protein expression (S4 Fig). Finally, we verified that all mutants were correctly localized at the PM, except Thi7^T80K^ and Thi7^S338L^, which were partially retained in the ER (S5 Fig).

The *thi7Δnrt1Δthi72Δthi4Δ* strain has impaired thiamine biosynthesis and transport. Therefore, this strain can be used to confirm whether the above mutants are deficient for thiamine transport by performing a phenotypic growth test. This strain was complemented with each Thi7 mutant and grown on solid medium containing a broad range of thiamine concentrations to determine which thiamine concentration sustains growth (Fig 4C, S6 Fig). As shown previously, *thi7Δnrt1Δthi72Δthi4Δ* cells grew only in the presence of the highest thiamine concentration (100 μM) because of unspecific thiamine entry. The growth of the *thi7Δnrt1Δthi72Δthi4Δ* strain complemented with Thi7^G59R^, Thi7^T80K^, Thi7^D85G^, Thi7^S130N^, Thi7^M247I^, Thi7^P286Q^, Thi7^T287N^, Thi7^P291Q^, Thi7^S338L^, Thi7^V398F^, or Thi7^A447P^ was restored at relatively low thiamine concentrations (10 nM), suggesting that their thiamine transport is sufficient to restore growth. The growth of the *thi7Δnrt1Δthi72Δthi4Δ* strain complemented with Thi7^N133K^ or Thi7^N350K^ was rescued at 1 μM thiamine, suggesting a much reduced thiamine transport (Fig 4C). Finally, the Thi7^M399R^ mutant was the most affected mutant and required at least 5 μM thiamine to restore growth of the *thi7Δnrt1Δthi72Δthi4Δ* strain (Fig 4C, S6 Fig). The inability of Thi7^N133K^, Thi7^N350K^, and Thi7^M399R^ mutants to sustain growth on low thiamine concentration is consistent with their strong resistance on oxythiamine-containing medium (Fig 4B).

We sought to gain insight into the structure of Thi7 to understand how these mutations could impact Thi7 transport. Thi7 belongs to the nucleobase-cation-symport-1 (NCS1) family of secondary active transporters, which includes the hydantoin transporter membrane hydantoin transport protein 1 (Mhp1) from *Microbacterium liquefaciens* [40]. The crystallized structure of Mhp1 displays 12 transmembrane helices. The transport mechanism, described by the alternating access model, implies structural changes from outward-facing (OF) to inward-facing (IF) conformations with different intermediate states adopted by the protein during the transition, among which at least one must be occluded [40–42]. Therefore, we generated multiple three-dimensional (3D) models of native and mutated Thi7. We modelized transporters in the OF open and occluded conformations as well as in the IF conformation. We also simulated thiamine docking within these models to predict the thiamine binding site (Fig 4D, S7 Fig). It appears that 8 mutants result from mutations of residues forming or surrounding the thiamine binding pocket: Thi7^G59R^, Thi7^D85G^, Thi7^N133K^, Thi7^M247I^, Thi7^P286Q^, Thi7^N350K^, Thi7^V398F^, and Thi7^M399R^ (Fig 4E). Therefore, the partial functionality of these mutants can be explained by disturbances localized in the binding pocket that prevent the interaction and stabilization of thiamine. For instance, this is directly visible for the mutants Thi7^N133K^ and Thi7^M399R^ (Fig 4F and 4G). The remaining mutants are localized outside of the binding pocket and could result in structural changes altering the stability of transmembrane spans and loops as well as the dynamics occurring from an OF to an IF state and therefore impacting transport. Any interaction with proteins or lipids could be affected.

### Thi7 mutants have impaired thiamine-induced endocytosis compared to WT Thi7

**Fig 5:**
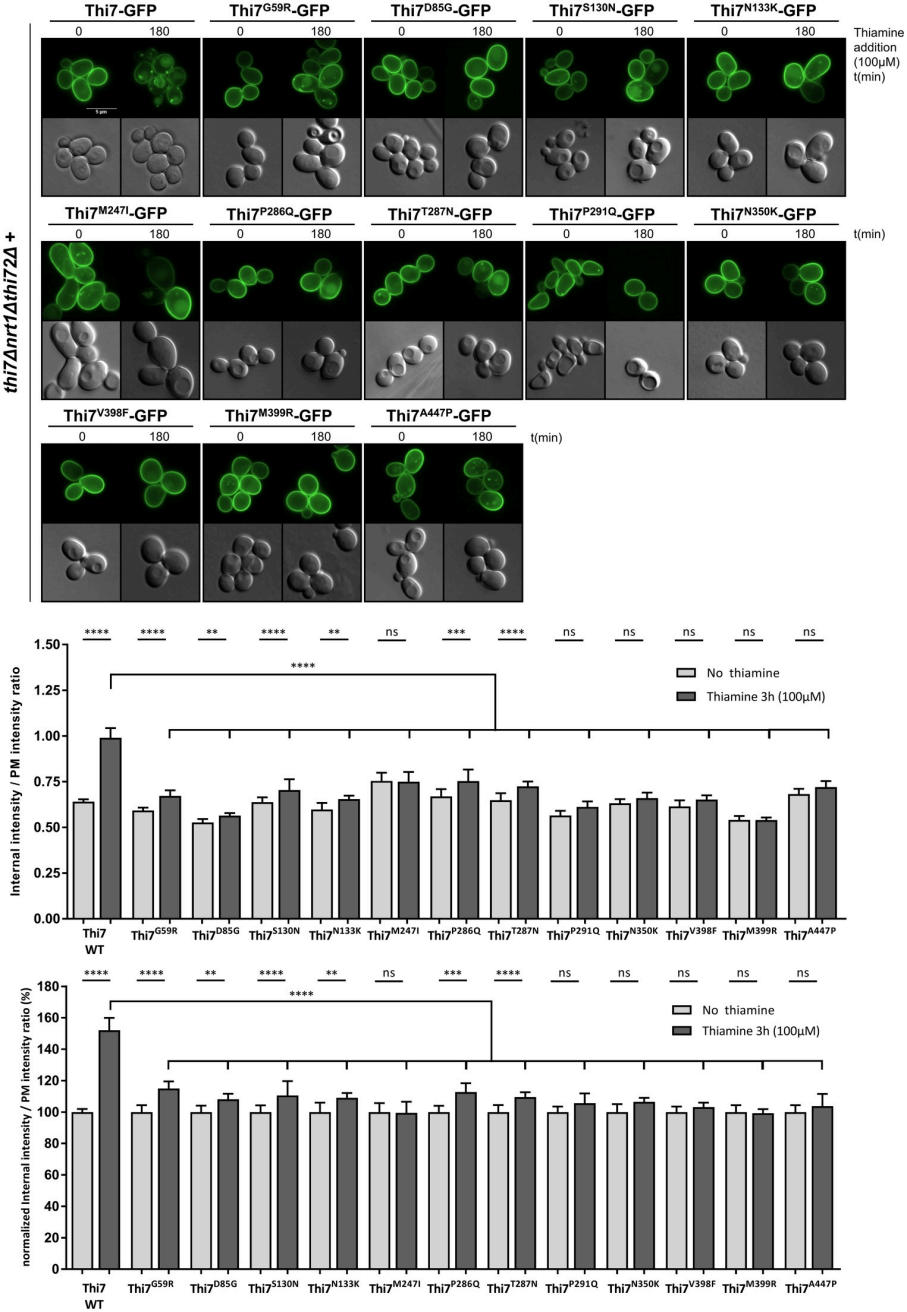
Single-point Thi7 mutants display thiamine-induced endocytic defects compared to WT Thi7. Localization of Thi7 mutants in a *thi7Δnrt1Δthi72Δ* strain after thiamine addition (final concentration: 100 μM) for 3 h into culture grown in thiamine-free selective medium. Scale bar represents 5 μm. Quantification shows the ratio of internal-over-PM fluorescence intensity of all single-point Thi7 mutants before and after thiamine addition (100 μM) as described in the Materials and methods section (*n* > 69 cells per condition in 3 replicates). The upper graph is data not normalized, whereas the bottom graph is data normalized for each mutant by the ratio at time 0. (****p < 0.0001; ***p < 0.001; **p < 0.01; *p < 0.05) The numerical data are included in S3 Data. GFP, green fluorescent protein; ns, nonsignificant; PM, plasma membrane; WT, wild type.

To correlate the partial functionality of these mutants with their endocytic behavior, we examined thiamine-induced endocytosis of the PM-localized Thi7 mutants in a *thi7Δnrt1Δthi72Δ* background. In thiamine-free medium, the Thi7 mutants are PM-localized with the appearance of a few intracellular punctate foci for Thi7^M247I^ and Thi7^A447P^ (Fig 5). After thiamine treatment, the majority of the mutants remained partially or totally within the PM compared to the WT Thi7, suggesting endocytosis defect (Fig 5). Thiamine-induced endocytosis of the most functionally affected mutants, Thi7^M399R^ and Thi7^N350K^, was totally abolished (Fig 5). Other mutants, including Thi7^D85G^ and Thi7^P291Q^, also resisted thiamine-induced endocytosis, as indicated by the low amount of intracellular signal after thiamine treatment.

### Thiamine uptake through Thi7^6KR^ is sufficient to trigger endocytosis of Thi7^M399R^ and Thi7^N350K^

**Fig 6:**
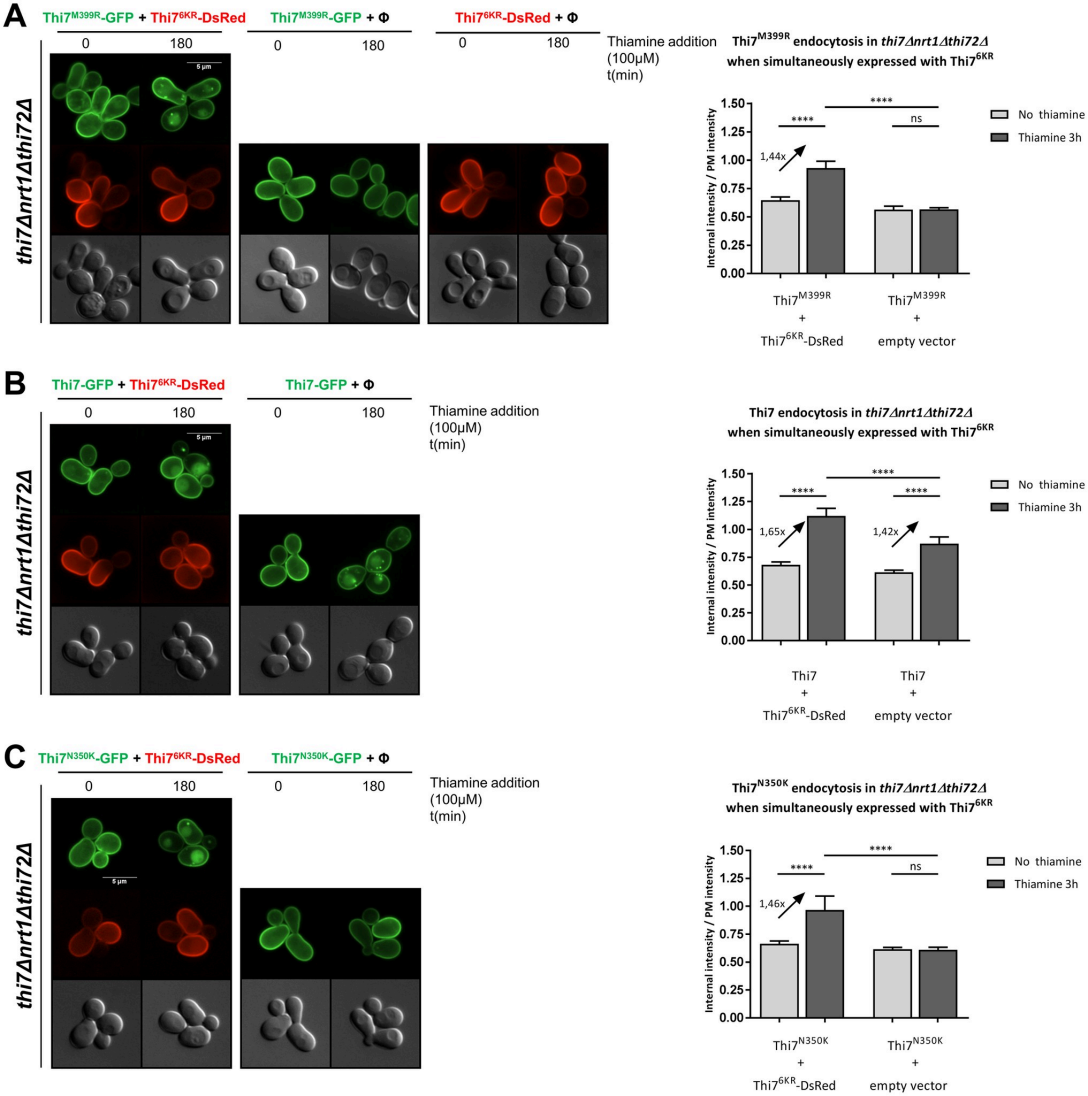
Thiamine uptake through Thi7^6KR^ leads to endocytosis of the inactive Thi7 transporters. (A) Localization of Thi7^M399R^-GFP and Thi7^6KR^-mDsRed after incubation with thiamine (final concentration: 100 μM) in a *thi7Δnrt1Δthi72Δ* strain coexpressing *THI7*^*M399R*^*-GFP* and *THI7*^*6KR*^*-mDsRed*, or *THI7*^*M399R*^*-GFP* with an empty vector (pRS313; Φ) or *THI7*^*6KR*^*-mDsRed* with an empty vector (pRS316; Φ). Relative quantification of the internal-over-PM fluorescence intensity ratio of Thi7^M399R^-GFP before and after incubation with thiamine (100 μM) when coexpressed with Thi7^6KR^-mDsRed or an empty vector (pRS313; Φ). (B) Localization of Thi7-GFP and Thi7^6KR^-mDsRed after incubation with thiamine (final concentration: 100 μM) in a *thi7Δnrt1Δthi72Δ* strain coexpressing *THI7-GFP* with an empty vector (pRS313; Φ) or *THI7-GFP* with *THI7*^*6KR*^*-mDsRed*. Relative quantification of the internal-over-PM fluorescence intensity ratio of Thi7^M399R^-GFP before and after incubation with thiamine (100 μM) when coexpressed with Thi7^6KR^-mDsRed or an empty vector (pRS313; Φ). (C) Localization of Thi7^N350K^-GFP and Thi7^6KR^-mDsRed after incubation with thiamine at a final concentration of 100 μM in a *thi7Δnrt1Δthi72Δ* strain coexpressing *THI7*^*N350K*^*-GFP* and either *THI7*^*6KR*^*-mDsRed or* an empty vector (pRS313; Φ). Relative quantification of the internal-over-PM fluorescence intensity of Thi7^N350K^-GFP before and after incubation with thiamine (100 μM) when coexpressed with either Thi7^6KR^-mDsRed or an empty vector (pRS313; Φ). For quantification, the median with 95% confidence interval is presented (*n* > 94 cells per condition in 3 biological replicates) (****p < 0.0001, Wilcoxon test). Scale bar represents 5 μm. The numerical data are included in S4 Data. GFP, green fluorescent protein; ns, nonsignificant; PM, plasma membrane.

Transporter endocytosis often relies on the accumulation of its substrate in the cytosol [26]. The resistance of the most functionally affected mutant Thi7^M399R^ to thiamine-induced endocytosis could be due to the low amount of thiamine in the cytosol. To address this question, we coexpressed Thi7^M399R^-GFP with the active but endocytosis-resistant Thi7^6KR^ mutant (fused to DsRed) in *thi7Δnrt1Δthi72Δ* cells. When solely expressed, Thi7^6KR^ and Thi7^M399R^ failed to be endocytosed upon thiamine treatment (Fig 6A). However, coexpression of Thi7^6KR^ and Thi7^M399R^ led to endocytosis of Thi7^M399R^ after thiamine treatment, whereas Thi7^6KR^ remained localized at the PM. Neither Thi7^M399R^ nor Thi7 appears to interact with Thi7^6KR^, as we did not detect vacuolar targeting of Thi7^6KR^ while endocytosed (Fig 6A and 6B). Similarly, although Thi7^N350K^ was functionally affected compared to the WT Thi7 and blocked at the cell surface when expressed alone, its coexpression with Thi7^6KR^ rescued its endocytosis (Fig 6C). Together, these results suggest that the increase of thiamine intracellular pool is necessary to induce Thi7 endocytosis in response to thiamine transport.

### Increased thiamine intracellular pool is not sufficient to down-regulate Thi7^D85G^ and Thi7^P291Q^

**Fig 7:**
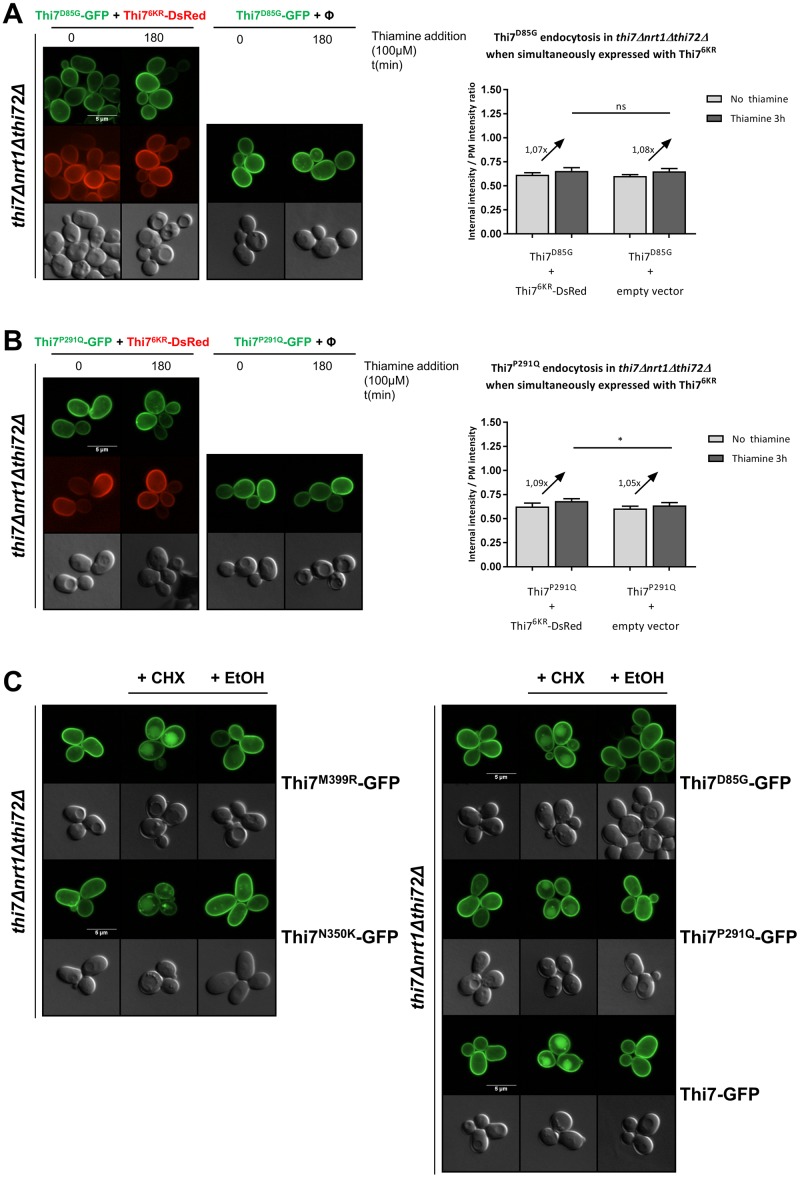
Uptake of thiamine through Thi7^6KR^ is not sufficient to induce endocytosis of Thi7^D85G^ and Thi7^P291Q^. (A) Localization of Thi7^D85G^-GFP and Thi7^6KR^-mDsRed after incubation with thiamine (final concentration: 100 μM) in a *thi7Δnrt1Δthi72Δ* strain coexpressing *THI7*^*D85G*^*-GFP* and either *THI7*^*6KR*^*-mDsRed* or an empty vector (pRS313; Φ). Relative quantification of the internal-over-PM fluorescence intensity of Thi7^D85G^-GFP before and after incubation with thiamine (100 μM) when coexpressed with either Thi7^6KR^-mDsRed or an empty vector (pRS313; Φ). (B) Localization of Thi7^P291Q^-GFP and Thi7^6KR^-mDsRed after incubation with thiamine (final concentration: 100 μM) in a *thi7Δnrt1Δthi72Δ* strain coexpressing *THI7*^*P291Q*^*-GFP* and either *THI7*^*6KR*^*-mDsRed* or an empty vector (pRS313; Φ). Relative quantification of the internal-over-PM fluorescence intensity of Thi7^P291Q^-GFP before and after incubation with thiamine (100 μM) when coexpressed with either Thi7^6KR^-mDsRed or an empty vector (pRS313; Φ). (****p < 0.0001; ***p < 0.001; **p < 0.01; *p < 0.05) (C) Localization of Thi7^M399R^-GFP, Thi7^N350K^-GFP, Thi7^D85G^-GFP, Thi7^P291Q^-GFP, and Thi7-GFP after incubation with CHX 50 μg/ml for 3 h or EtOH in a *thi7Δnrt1Δthi72Δ* strain grown in thiamine-free medium. For quantification, the median with 95% confidence interval is presented (*n* > 101 cells per condition in 3 biological replicates) (*p < 0.05; Wilcoxon test). Scale bar represents 5 μm. The numerical data are included in S5 Data. CHX, cycloheximide; EtOH, ethanol; GFP, green fluorescent protein; KR, lysine-to-arginine; ns, nonsignificant; PM, plasma membrane.

Thi7^M399R^ and Thi7^N350K^ resist thiamine-induced endocytosis because of the absence of intracellular accumulation of thiamine. Yet, Thi7^D85G^ and Thi7^P291Q^, individually expressed, also resist thiamine-induced endocytosis although they seem to still transport thiamine, albeit with reduced functionality (Figs 4C, 5, 7A and 7B). Even when coexpressed with the active Thi7^6KR^-DsRed in *thi7Δnrt1Δthi72Δ* cells, which fully restores the uptake of thiamine, no endocytosis was observed for these mutants (Fig 7A and 7B). Hence, in contrast to Thi7^M399R^ and Thi7^N350K^ mutants, the impaired thiamine-induced endocytosis of Thi7^D85G^ and Thi7^P291Q^ cannot be explained by only the lower intracellular pool of thiamine resulting from their reduced transport activity. Rather, these data suggest that these mutants adopt a specific conformation that might mask putative binding sites required for ubiquitylation and/or endocytosis. Interestingly, CHX treatment induces endocytosis of Thi7^M399R^ and Thi7^N350K^ as well as Thi7^D85G^ and Thi7^P291Q^ mutants (Fig 7C), suggesting another thiamine-independent pathway involved in the degradation of Thi7.

### Thiamine-induced endocytosis of Thi7 requires TORC1 and Sit4 but is not inhibited by Npr1

**Fig 8:**
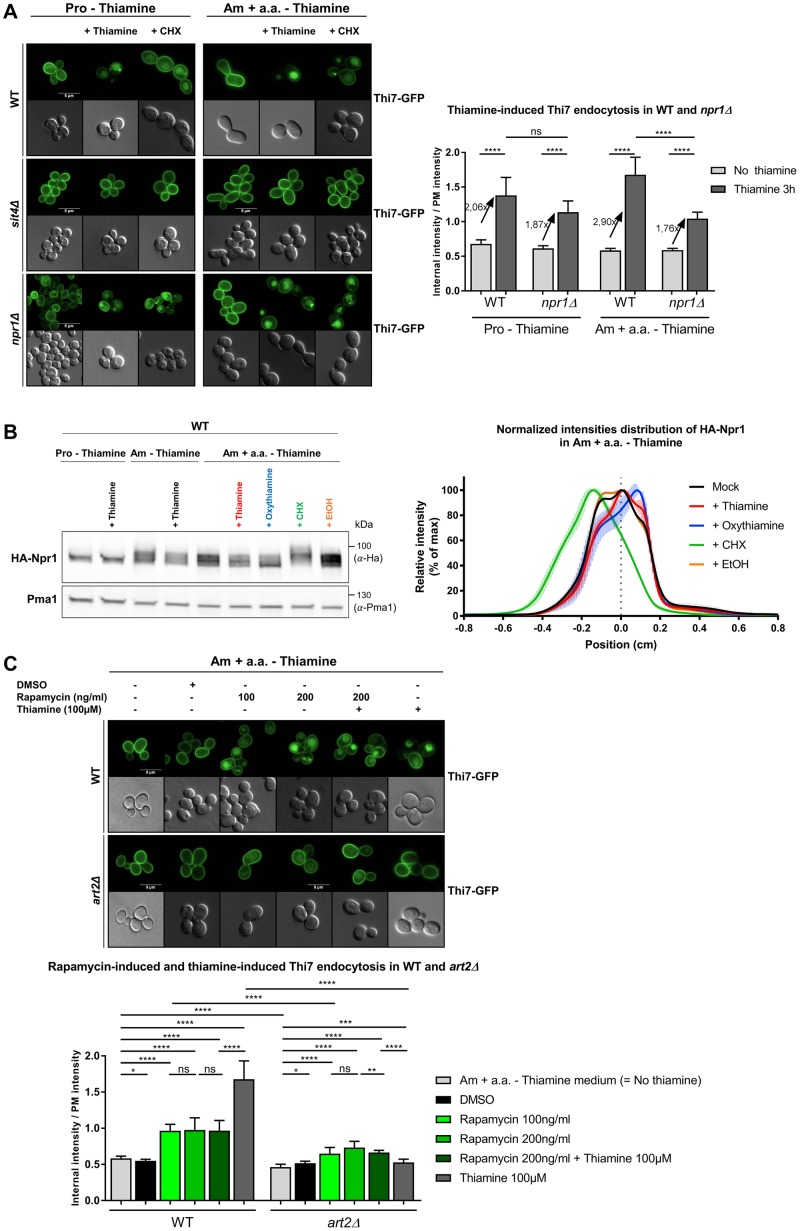
Thiamine-induced endocytosis of Thi7 appears to require TORC1 and Sit4 but is not inhibited by Npr1. (A) Localization of Thi7-GFP in WT, *sit4Δ*, and *npr1Δ* strains complemented with the pFL36-HIS3-LYS2 or pFL36-HIS3-MET15 plasmid grown in thiamine-free medium containing proline (10 mM) as the sole nitrogen source (“Pro–Thiamine”) or on ammonium-(38 mM) containing thiamine-free complete medium (“Am + a.a.–Thiamine”). Cells were incubated for 3 h with or without thiamine (100 μM) or CHX (50 μg/ml). Relative quantification of the internal-over-PM fluorescence intensity of Thi7-GFP in the different conditions (*n* > 52 cells). (B) Addition of thiamine or oxythiamine leads to de-phosphorylation of HA-Npr1. A WT strain expressing *HA-NPR1* and complemented with the pFL36 plasmid was grown up to early log phase in thiamine-free medium containing proline (10 mM) as the sole nitrogen source and incubated or not for 3 h with thiamine (100 μM), on thiamine-free medium containing ammonium (38 mM) as sole nitrogen source and incubated for 3 h with or without thiamine (100 μM) and ammonium-containing thiamine-free complete medium incubated for 3 h with thiamine (100 μM), oxythiamine (100 μM), CHX (50 μg/ml), or EtOH before being harvested. Cell extracts were immunoblotted with anti-HA and anti-Pma1 antibodies. Normalized intensities; distribution of HA-Npr1 in ammonium-containing thiamine-free complete medium with or without incubation with thiamine 100 μM, oxythiamine 100 μM, CHX 50 μg/ml, or EtOH. (C) Localization of Thi7-GFP in WT and *art2Δ* strains grown in ammonium-containing thiamine-free complete medium (“Am + a.a.–Thiamine”) and incubated for 3 h with or without DMSO, rapamycin 100 ng/ml, rapamycin 200 ng/ml, rapamycin 200 ng/ml + thiamine 100 μM, or thiamine 100 μM. Relative quantification of the internal-over-PM fluorescence intensity of Thi7-GFP in the different conditions. For quantification in (A) and (C), the median with 95% confidence interval is presented (*n* > 32 cells). Scale bar represents 5 μm (****p < 0.0001; ***p < 0.001; **p < 0.01; *p < 0.05). The numerical data are included in S6 Data. CHX, cycloheximide; EtOH, ethanol; GFP, green fluorescent protein; HA, hemagglutinin; ns, nonsignificant; PM, plasma membrane; Pma1, plasma membrane ATPase1; TORC1, target of rapamycin complex 1; WT, wild type.

Results within this study suggest that thiamine-induced endocytosis of Thi7 is Art2 dependent. Although increased intracellular pool of thiamine partially explains Thi7 endocytosis in response to thiamine transport, how Art2-mediated ubiquitylation is stimulated by thiamine remains unclear. In the current model, transporter endocytosis often relies on the activation of α-arrestin via dephosphorylation [13,19,20,23]. The TORC1-Npr1-Sit4 pathway was shown to regulate Art1 and Bul α-arrestins in response to variations in nitrogen availability [29]. Moreover, CHX, which stimulates TORC1 activity, induces Art2-mediated endocytosis of thiamine transporters in the absence of thiamine. Therefore, we tested the role of this pathway in Thi7 endocytosis (Fig 8). In the *sit4Δ* strain grown in thiamine-free proline medium (low TORC1 activity) as well as thiamine-free ammonium-containing complete medium (high TORC1 activity), both the CHX- and thiamine-induced endocytosis of Thi7 was inhibited, indicating that the two processes are Sit4 dependent (Fig 8A). If thiamine-induced endocytosis of Thi7 is inhibited by Npr1, the deletion of *NPR1* should lead to constitutive Thi7 endocytosis. Such a phenotype was observed for Gap1 in the *npr1Δ* mutant grown in proline medium and resulted from permanently dephosphorylated Bul1/2 [13]. However, the deletion of *NPR1* did not lead to constitutive endocytosis of Thi7, which remains sensitive to CHX- and thiamine-induced down-regulation (Fig 8A). This suggests that Thi7 endocytosis is Npr1 independent. Yet, the rate of thiamine-induced endocytosis of Thi7 was higher in the WT than in the *npr1Δ* mutant, especially in thiamine-free ammonium-containing complete medium, suggesting that thiamine has a positive influence on the Npr1 kinase and subsequently on Thi7 endocytosis. Therefore, we examined the consequence of thiamine on Npr1 phosphorylation profile. Previous studies have shown that TORC1 activation leads to hyperphosphorylation and subsequent inactivation of Npr1 [44]. In WT cells grown in thiamine-free proline medium, the hemagglutinin (HA)-Npr1 construct migrated as two close bands below 100 kDa, regardless of thiamine presence. When cells were grown in ammonium-containing thiamine-free complete medium, HA-Npr1 is phosphorylated. Interestingly, addition of thiamine or oxythiamine led to apparent dephosphorylation of HA-Npr1, although only observable after 3 h of thiamine addition and not at early time points (Fig 8B, S8 Fig). In this growth medium, CHX treatment led to an upward shift to the HA-Npr1 protein, indicating a higher level of phosphorylation. Together, those results are consistent with the idea that thiamine might dephosphorylate Npr1, stimulate Art2, and induce Thi7 endocytosis in response to transport. Finally, we tested the influence of rapamycin, an inhibitor of TORC1 activity, on Thi7 localization. Previous studies reported that several permeases such as Gap1, Can1, lysine-specific permease 1 (Lyp1), Fur4, and Jen1 were endocytosed upon rapamycin treatment, although the underlying mechanisms of this stress-induced down-regulation remain poorly understood [21,45]. In thiamine-free medium, rapamycin treatment (100 and 200 ng/ml) induced Art2-dependent endocytosis of Thi7, yet the endocytosis rate was similar regardless of rapamycin concentration and always lower than the one observed upon thiamine addition (Fig 8C). A simultaneous treatment with rapamycin and thiamine also led to a lower endocytosis rate of Thi7, corresponding to the rate of rapamycin treatment alone. With the rate of Thi7 endocytosis being similar upon rapamycin treatment, regardless of thiamine addition, we suggest that the observed endocytosis was due to rapamycin only and that the thiamine-induced pathway that initiates Thi7 endocytosis was partially inhibited by rapamycin. Therefore, our data suggest that the thiamine-induced pathway could be TORC1 dependent. Taken together, these results indicate that thiamine-induced endocytosis of Thi7 requires the Sit4 phosphatase and might be TORC1 dependent. Interestingly, Npr1 does not inhibit Art2-dependent endocytosis. Our data rather suggest a positive role of Npr1 on Thi7 endocytosis.

## Discussion

We developed an innovative approach to explore the high complexity of the α-arrestin/PM protein system using quantitative proteomics. Our proteomic analysis led to the identification of 33 new potential targets of the α-arrestin family. CHX was used as a global endocytosis-triggering stimulus in order to study α-arrestin dependency on protein trafficking without focusing on their specific substrates. Indeed, CHX treatment is frequently used to trigger endocytosis of PM proteins in a substrate-independent manner [6,7,19]. Additionally, CHX treatment allowed us to observe transporter–α-arrestin interactions that are in line with recent publications, which indirectly supports our mass-spectrometry methodology. Nevertheless, the use of CHX also has limitations and could produce indirect effects affecting protein turnover. Undeniably, the protein translation inhibition by CHX could affect the abundance of PM proteins, yet it allowed us to focus on proteins existing before the treatment and avoid biasing the analysis with newly synthesized proteins. Nevertheless, we cannot rule out that it may give rise to additional regulations and false positive variations. For example, we could expect that proteins with a very long half-life (i.e., a low turnover rate) will be less endocytosed during the 90 min of CHX treatment and thereby less represented in our screening. Nevertheless, 33 proteins were identified as affected by CHX treatment out of the 93 proteins identified by the screening, including proteins with a long half-life, such as Pma1 (about 11 h) [46] and Pdr5 (60–90 min) [47]. Moreover, the fast CHX-dependent depletion of the high-turnover-rate proteins from the PM could lead to a proportional increase of the abundance of the remaining low-turnover-rate proteins. Nevertheless, we found that, despite its very low turnover rate, Pma1 abundance after CHX treatment of a WT strain decreased to 68% of its value after mock treatment (Table 1), supporting the idea that this proportional increase is weak compared to the decrease triggered by CHX. In our screening, only a few proteins were more abundant upon CHX treatment; part of this increase might be explained by such effect. Regarding the *artΔ*+CHX/WT+CHX ratios, the same CHX treatment was applied to both strains. Therefore, the effect of CHX on the PM landscape should be mostly comparable in each sample, and we suspect that the remaining differences of PM protein composition are specific to the α-arrestin deletion.

The results from our screening support the view that several adaptors can be involved in the endocytosis of one specific PM protein (Table 2). This phenomenon indicates that the functions of the various α-arrestins are not redundant. Indeed, the fact that several single α-arrestin gene deletions independently affect the abundance of PM proteins suggests that these adaptors may work in an additive manner in endocytosis. In addition, it could be that some α-arrestins are involved in other trafficking steps between intracellular compartments (endosomes, Golgi apparatus, multivesicular body [MVB] sorting). For instance, Art4 has been proposed to act at the Golgi apparatus, consolidating the fate of the lactate transporter Jen1 en route to the vacuole by additional ubiquitylation [48]. Moreover, it cannot be excluded that some α-arrestins could function in trafficking as oligomeric complexes. The screening also revealed that in the absence of α-arrestins, the abundance of several PM proteins was decreased. This decrease could result from a global modification of gene expression due to cellular adaptation to α-arrestin deletion or transporter stabilization within the PM. In addition, this decreased abundance could be directly connected to the function of some α-arrestins in the recycling of endocytosed proteins from internal compartments to the PM. In this case, the lack of such adaptors would increase the proportion of proteins targeted for vacuolar degradation. For instance, Art3/6 are believed to promote the retrograde transport of the general amino acid permease Gap1 from endosomes to the Golgi apparatus [49], which could lead to Gap1 recycling at the PM.

Our screening highlights for the first time, to our knowledge, a link between CHX treatment and the abundance of PM proteins such as a GTP-binding protein (Ras2), components of eisosomes (Nce102), the ER–PM tethering system (Ist2, Tcb1/3), and the cell integrity pathway (Rho1, Zeo1). These proteins were more abundant in response to CHX treatment in the WT strain. We hypothesize that proteins involved in the maintenance of PM integrity are rapidly recruited to the PM in order to assist cells with the modifications in cell surface protein and lipid composition induced by CHX treatment. The reduced abundance of Fks1 and Gas1 (involved in cell wall synthesis and maintenance) after CHX treatment is consistent with this hypothesis. Eisosomes laterally organize the PM by forming sphingolipids and ergosterol-enriched membrane compartments containing Can1 (MCCs), in which Nce102 acts as a sphingolipid sensor [50,51]. The CHX treatment of cells could therefore have an impact on the stability of eisosomes and stimulates endocytosis of transporters. Indeed, the clustering of Can1 and Fur4 in these MCCs prevents their α-arrestin-dependent ubiquitylation and endocytosis [52,53].

The current proteomic study led to the identification of thiamine transporters as new targets of the α-arrestin family. Our results support a common regulation of the endocytosis of Thi7 and two highly similar proteins, the putative low-affinity thiamine transporters Nrt1 and Thi72. It appears that Art2 α-arrestin is the main mediator of CHX- and thiamine-induced Rsp5-dependent ubiquitylation and endocytosis of thiamine transporters. This situation is reminiscent of the closely related uracil permease Fur4, whose substrate- and CHX-induced endocytosis is mediated by the same α-arrestins, Art1/2/8 and Bul1/2 [7]. Although the screening results suggested an effect of Art9 in Thi7 endocytosis, no impairment of Thi7 endocytosis by *ART9* deletion was detected by immunoblotting and fluorescence microscopy. We verified that the quantification data generated by the proteomic analysis were not biased by common peptides that are shared between Thi7, Nrt1, and Thi72. The quantitative effect of Art9 on Thi7 remains unexplained. We hypothesize that a difference in the growth conditions could be responsible of this effect. Indeed, native Thi7 could interact with the low-affinity transporters and be endocytosed with the latter in an Art9-dependent manner in the screening conditions (i.e., YD medium). Nevertheless, there is no evidence in the literature suggesting any (hetero-)oligomerization of thiamine transporters, and neither Thi7^M399R^-GFP nor Thi7-GFP appears to interact with Thi7^6KR^-DsRed in our study (Fig 6), suggesting that Thi7 itself does not form homo-oligomers. In addition, we have no indication about the expression patterns of the three transporters in YD medium. Therefore, the probability of oligomerization and the stoichiometry of this interaction remain unclear but cannot be ruled out.

In order to investigate the underlying mechanism of Thi7 endocytosis in response to thiamine transport, we isolated transport-defective mutants and examined their thiamine-induced endocytosis. Six mutations that our modeling experiments localized in the binding pocket of thiamine (Fig 4E) had no observable oxythiamine transport: Thi7^G59R^, Thi7^D85G^, Thi7^N133K^, Thi7^P286Q^, Thi7^N350K^, and Thi7^M399R^. However, growth tests as readout of thiamine transport indicated that thiamine uptake was the most defective in Thi7^N133K^, Thi7^N350K^, and Thi7^M399R^. The two phenotypic growth tests used in this study do not have the same sensitivity. For instance, our results suggest that Thi7^G59R^, Thi7^D85G^, and Thi7^P286Q^ transport thiamine but not oxythiamine. Transporter affinity for thiamine and oxythiamine could be slightly different, although the two molecules are highly similar. We believe that Thi7^G59R^, Thi7^D85G^, and Thi7^P286Q^ are functionally defective for thiamine transport, but the current assay is not sensitive enough to detect this. Indeed, TPP is a coenzyme rather than a metabolite, and cells require very low amounts of it to maintain metabolism and growth. As a consequence, this assay only detects mutants with severe transport defect. Consistent with this idea, the strain containing Thi7^T287N^ rescued growth even at low thiamine concentrations, yet Thi7^T287N^ transport has been shown to be only 20%–25% of that of WT Thi7 [39].

In addition, we provide evidence that the intracellular accumulation of thiamine is required to induce endocytosis of Thi7 in response to thiamine transport. Each Thi7 mutant was partially functional and displayed thiamine-induced endocytic defects compared to WT Thi7. Moreover, Thi7^N350K^ and Thi7^M399R^ (most functionally affected mutants) resisted thiamine-induced endocytosis, but thiamine uptake by coexpression of Thi7^6KR^ fully rescued their endocytosis. These were the first set of results that link reduced thiamine accumulation to decreased endocytosis. Oxythiamine triggers Thi7 endocytosis with similar kinetics as thiamine. Internalized oxythiamine is phosphorylated by the thiamine pyrophosphokinase Thi80, and phosphorylated oxythiamine binds to and inhibits TPP-dependent enzymes [54]. This suggests that TPP-dependent enzyme activity is not essential for Thi7 endocytosis. Our results also suggest that internal thiamine, although required to induce Thi7 endocytosis in response to transport, is not sufficient per se. Indeed, the impaired endocytosis of Thi7^D85G^ and Thi7^P291Q^ cannot only be explained by reduced transport activity and absence of substrate accumulation, since restoration of thiamine uptake failed to rescue endocytosis. One explanation for this phenotype is that Thi7^D85G^ and Thi7^P291Q^ interact with Thi7^6KR^ and alter its transport activity, resulting in lower thiamine accumulation and resistance to endocytosis. Nevertheless, no interaction has been identified between Thi7^6KR^ and WT Thi7, Thi7^N350K^, or Thi7^M399R^. Moreover, we did not observe any growth defect of cells expressing *THI7*^*KR*^ mutants alone or with transport-deficient mutants, suggesting that thiamine transport is sufficient to sustain growth. Finally, expression of *THI7*^*KR*^ in a *thi7Δnrt1Δthi72Δ* strain deficient for thiamine transport led to impaired growth on oxythiamine-containing medium (Fig 3), whereas it sustains growth on medium containing low amounts of thiamine. Therefore, we rather speculate that these mutants are blocked in a conformation that conceals a putative Art2-interaction domain required for ubiquitylation and endocytosis. The residue P291 does not appear to be involved in the substrate binding site, and the P291Q mutation could rather result in structural changes in the seventh transmembrane span (TM7) or affect the cytosolic loop between TM6 and TM7. Recent studies identified transport-elicited conformational changes that are required to down-regulate Can1 and Fur4 in response to transport [26,29]. By contrast, the conformation of Thi7^M399R^ and Thi7^N350K^ would permanently expose the α-arrestin-targeted domain, allowing endocytosis to occur as a result of thiamine uptake by Thi7^6KR^. Although the conformation of these mutants within the PM is unknown, Thi7^N350K^ and Thi7^M399R^ might be blocked in an IF state, whereas Thi7^D85G^ and Thi7^P291Q^ would be in an OF state. In the latter, an OF-to-IF switch that unmasks a degron essential for endocytosis would be inhibited. A clue suggesting the existence of a degron in Thi7 comes from its similarity with Fur4 (28% of sequence identity). Like Fur4, Thi7 has a loop-interaction domain (LID) localized in the cytosolic C-terminal tail close to lysine residues essential for ubiquitylation. The LID sequence of Fur4 was shown to interact with cytosolic loops, acting like a conformational sensor [26]. The disruption of the LID-loops interaction would expose a degron targeted by the ubiquitin ligase Rsp5. Even so, we cannot rule out that endocytosis of Thi7 may not require the exposure of such degron. Indeed, its endocytosis upon CHX treatment occurs without thiamine transport catalysis and a priori no structural rearrangement. On the one hand, CHX-induced Thi7 endocytosis could rather be explained by a direct impact of CHX on Thi7 activity and/or on membrane integrity. Alternatively, CHX may have a direct impact on the exposure of the Art2-binding site/degron without thiamine uptake either by inducing posttranslational modifications of Thi7 or by affecting its lipid environment. Additionally, TORC1 stimulation by CHX may also give rise to posttranslational modifications of Thi7 and lead to structural rearrangements. Indeed, a phosphoproteomic study suggested that the phosphorylation status of the C-terminal tail of Thi7 is affected by rapamycin [55]. Moreover, a serine and a threonine residue of the C-terminal tail are predicted to be localized inside a casein kinase 1 complex (Yck1/2) recognition motif, which is reminiscent of Yck1/2-dependent phosphorylation required for Fur4 endocytosis [56,57]. In addition, the clustering of Can1 or Fur4 in the sphingolipid-enriched membrane compartments is linked to its conformation and protects it from ubiquitin-dependent endocytosis [52,53]. CHX could trigger the exclusion of Thi7 from such a domain, affect its conformation, and lead to the exposure of the Art2-binding site without thiamine transport catalysis. Additional work will be required to confirm and identify this degron. Interestingly, Thi7^D85G^ and Thi7^P291Q^ are resistant to thiamine-induced endocytosis but are still endocytosed upon CHX addition. These results could be explained by a different posttranslational status of Art2 after CHX and thiamine treatment. Many phosphorylation sites were identified on Art2, but only a few depend on the TORC1 pathway [58]. Therefore, the difference between CHX- and thiamine-induced endocytosis may be also due to differential phosphorylation status of these residues.

We have shown that thiamine- and CHX-induced endocytosis of WT Thi7 is Art2 and Sit4 dependent. However, strong evidence is still lacking in this study to confirm the role of Npr1 and TORC1 in thiamine-induced endocytosis of Thi7. We showed that thiamine-induced endocytosis of Thi7 is partially inhibited by rapamycin and could therefore be TORC1 dependent (Fig 8C). Our data might be difficult to interpret, as rapamycin alone induces Thi7 endocytosis via an unknown pathway. Thus, the inhibition of thiamine-induced Thi7 endocytosis by rapamycin could rather be explained as follows: as rapamycin induces Thi7 endocytosis, thiamine uptake is reduced and impacts the rate of Thi7 endocytosis as less thiamine is accumulated in the cells. Nevertheless, thiamine and rapamycin were added simultaneously, and thiamine uptake is known to be a fast process [59]. We suspect thus that the thiamine uptake required to trigger endocytosis takes place early enough to not be affected by the rapamycin-induced removal of Thi7 from the PM. For this reason, we rather believe that the thiamine-induced pathway that initiates Thi7 endocytosis is partially inhibited by rapamycin and could be TORC1 dependent. Consistent with this, although the possibility of a TORC1-independent Sit4 involvement in Thi7 endocytosis is attractive, we did not find clear evidence in the literature of Sit4-dependent, TORC1-independent regulatory processes. Concerning the role of Npr1 kinase in Thi7 endocytosis, we detected some changes in Npr1 phosphorylation profile upon thiamine treatment but only after 3 h of treatment and not at early time points (S8 Fig). Moreover, thiamine-induced endocytosis of Thi7 is poorly affected by the deletion of *NPR1*, which rather suggests that Npr1 plays a minor role in regulation of Thi7 endocytosis (Fig 8A). Therefore, we suspect that other kinases may be involved in addition to Npr1. The weak effect of *NPR1* deletion on Thi7 endocytosis could be explained by an increased activity of other kinases. Indeed, the Npr1 phosphorylation profile is not the only readout of TORC1 activity. Since our data suggest that Npr1 phosphorylation is modulated by the presence of thiamine, we could also even speculate that Npr1 is slightly positively regulating Thi7 endocytosis if we assume that phosphorylation status is correlated to the activity of Npr1. Consistent with this, previous studies have shown that Npr1 stimulates endocytosis of Bap2 (leucine) and Tat2 (tryptophan) transporters by phosphorylating directly the transporter [44,59]. Alternatively, Npr1 could also directly modify the conformation or activity of Thi7 as previously discovered for the ammonium transporter Mep2 [60]. Modulation of Thi7 conformation or activity would, in turn, modify its endocytosis rate. Regarding the regulation of Thi7 endocytosis by CHX, our results do suggest that CHX-induced endocytosis of Thi7 is TORC1 dependent. Indeed, Npr1 is highly phosphorylated in response to CHX addition, even 3 h after treatment, at a concentration that triggers Thi7 endocytosis. Moreover, the proportion of Thi7 targeted to the vacuole upon CHX treatment is lower in WT cells grown in medium containing proline as the sole nitrogen source, which is known to keep TORC1 poorly active [44], compared to those grown in ammonium-containing complete medium (Figs 1 and 8A). The low TORC1 activity could dampen the TORC1-stimulating effect of CHX, supporting the idea that CHX-induced endocytosis of Thi7 is TORC1 dependent.

**Fig 9:**
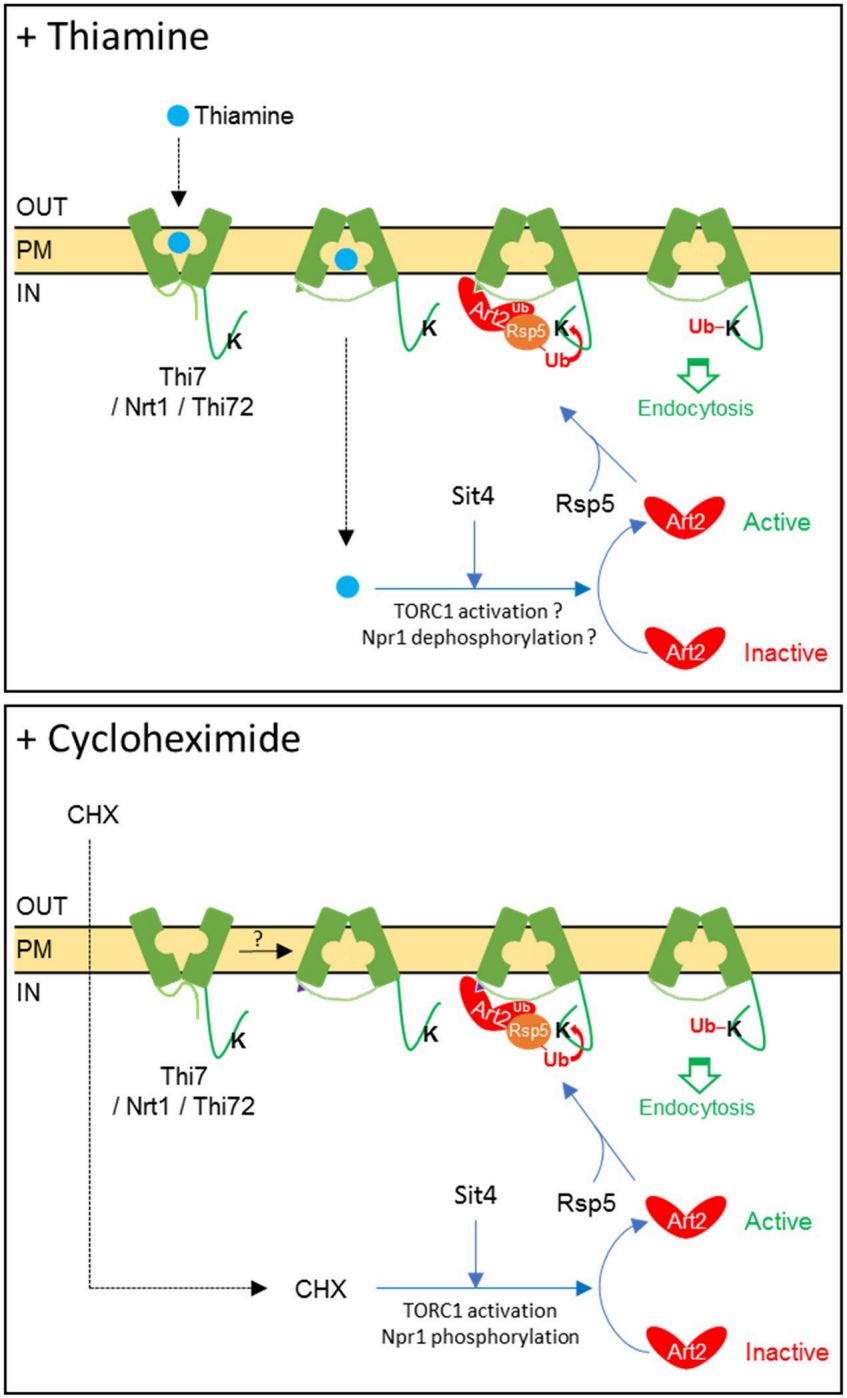
Model of Art2-dependent ubiquitylation and endocytosis of Thi7, Nrt1, and Thi72. (Top panel) In the absence of thiamine, Art2 is inactive. Thiamine uptake through Thi7, Nrt1, or Thi72 induces conformational changes, leading to the exposure of an Art2-binding domain. Thiamine accumulation within the cytosol leads to Art2-mediated ubiquitylation of thiamine transporters. This process requires the Sit4 phosphatase, and our results also suggest an active TORC1 and a poorly phosphorylated version of Npr1. (Bottom panel) Alternatively, CHX diffuses through the cell membrane and stimulates TORC1. CHX-activated TORC1 leads to hyperphosphorylation and inactivation of Npr1. CHX treatment induces Art2-dependent endocytosis of thiamine transporters, which also depends on the Sit4 phosphatase. In the absence of thiamine transport, no conformational change occurs on the transporter. CHX, cycloheximide; K, Rsp5-targeted lysine residue(s); Nrt1, nicotinamide riboside transporter 1; PM, plasma membrane; TORC1, target of rapamycin complex 1; Ub, ubiquitin.

Taken together, our results suggest a new model for Art2-dependent ubiquitylation and endocytosis of thiamine transporters (Fig 9). This model proposes that thiamine supply controls the abundance of thiamine transporters in a TORC1-dependent manner. We have provided evidence that the phosphatase Sit4 is involved in Art2-dependent Thi7 endocytosis upon thiamine and CHX treatment. However, our data do not support an inhibitory role of Npr1 in thiamine-induced pathway. Therefore, additional work will be required to describe in detail all the facets of Npr1-dependent regulation of α-arrestins and thiamine transporters.

## Materials and methods

### Yeast strains and growth conditions

Yeast strains used in this study (S3 Table) are derivatives of *Saccharomyces cerevisiae* auxotrophic strain BY4742. The *npi1–1* (*rsp5*) mutant strain, obtained by insertion of a selective marker in the *RSP5* promoter and resulting in an attenuated *RSP5* gene expression by a factor of 10, was provided by Pr. Bruno André (ULB, Belgium). The *art2Δart9Δ* strain was obtained by yeast crossing and tetrad dissection. The *end3Δart2Δ* strain was generated by homologous recombination using a *natNT2* deletion cassette on an *end3Δ* background. The strains lacking genes encoding the thiamine transporters Thi7, Nrt1, and Thi72 and the gene encoding the thiazole synthase Thi4 were received from Pr. Jürgen Stolz (TUM, Germany). For proteomic analysis, yeast strains were grown in YD medium (2% glucose and 2% KAT yeast extract). For Thi7 endocytosis experiments, transformed yeast strains were grown in selective thiamine-free complete medium (2% glucose, 0.69% yeast nitrogen base [YNB] without amino acids and thiamine [ForMedium, Norfolk, United Kingdom], Drop-Out powder, supplemented with required amino acids [pH 5.2]). To study the influence of thiamine on TORC1/Npr1/Sit4 pathway, yeast strains were grown in minimal medium (2% [w/v] glucose, 10 mM proline [Sigma-Aldrich, Schnelldorf, Germany] as unique nitrogen source, 0.19% [w/v] YNB without amino acids, ammonium sulfate and thiamine). In case of ammonium-containing medium, 0.69% (w/v) YNB without amino acids and thiamine was used.

### Plasmids

The plasmids used in this study are listed in S4 Table. We received from Pr. Jürgen Stolz (TUM, Germany) a high-copy plasmid expressing *THI7-GFP* under the control of the *THI7* promoter from which we transferred the cassette into a pRS316 centromeric plasmid. Plasmids encoding N- and C-terminally GFP-fused versions of Thi7, Nrt1, and Thi72 were designed using Gibson assembly technology (NEB) [61]. KR mutants of *THI7* were engineered using synthetic oligonucleotides (Integrated DNA Technologies, Leuven, Belgium) and Gibson assembly. Plasmids encoding monomeric DsRed-fused versions of thiamine transporters were designed using Gibson assembly. The plasmid expressing *3xHA-ART2* under control of the endogenous promoter was provided by Pr. Hugh Pelham (MRC, UK) and is based on the YCplac111 vector. In this study, THI7-GFP mutants were isolated from an oxythiamine-based screening. All these genes are controlled by the *THI7* promoter and the *ADH1* terminator. The QuikChange site-directed mutagenesis method was used to design *THI7*^*T287N*^*-GFP*. All plasmids were verified by sequencing.

### Oxythiamine-based screening

The *thi7Δnrt1Δthi72Δ* strain expressing *THI7-GFP* was grown overnight in a thiamine-free medium, plated on solid medium containing oxythiamine 3 μM and 6 μM, and grown for 3 days, and 156 spontaneous growing colonies were analyzed by epifluorescence microscopy based on protein expression and localization. Plasmids were extracted, purified in *Escherichia coli*, sequenced, and retransformed into the *thi7Δnrt1Δthi72Δ* and *thi7Δnrt1Δthi72Δthi4Δ* strains for characterization.

### Proteomic analysis

WT and 12 knockout strains (*artΔ*) were grown at 28 °C to an optical density (OD_600nm_) of 7 (3 × 10^7^ cells/ml) in 2 L of YD. Cultures of the 12 *artΔ* strains were incubated with CHX (Sigma-Aldrich, Schnelldorf, Germany) (solubilized in 100% ethanol) at a final concentration of 50 μg/ml for 90 min. As a control, an additional WT strain was mock treated with the same volume of 100% ethanol. The final concentration of ethanol in the medium was 0.5%. Cells were harvested and homogenized, and PM enrichment was performed. The PM enrichment protocol was previously developed in our laboratory [62–65]. Twenty micrograms of proteins were digested with trypsin and labeled with isobaric tags for absolute and relative quantification (iTRAQ, ABSciex) as previously described [63]. Labeled peptides were resuspended in 78 μl of a 2% (v/v) acetonitrile (ACN) and 0.1% (v/v) formic acid solution in order to obtain a final concentration of 2 μg/μl. Peptide separation was performed on an ultrahigh-performance liquid chromatography (UHPLC) (Eksigent 2D nano-ultra, Sciex). Two micrograms of labeled peptides were diluted in 5 μl of a 2% ACN–0.5% formic acid solution and injected on a C18 pre-column (Acclaim PepMap 100 C18, 2 cm, 100 μm, Thermo Scientific). Eluted peptides were immediately subjected to reverse phase (RP) chromatography on a C18 analytical column (Acclaim PepMap 100 C18, 75 μm id × 25 cm, Thermo Scientific) for 150 min at a flow rate of 300 nl/min using a gradient of ACN. The iTRAQ-labeled peptides were analyzed by electrospray ionization (ESI) TripleTOF 5600+ System (AB Sciex). The experiment was performed with three biological replicates of each *artΔ* strain and six biological replicates of the CHX- and mock-treated WT.

Peptide identification and quantification were performed using ProteinPilot 5.0 software, Paragon search algorithm (AB Sciex), and the *S*. *cerevisiae* UniProtKB protein database. The PM proteome of the CHX- treated WT strain was compared with the one of the mock-treated WT strain to select PM proteins whose abundance is affected upon CHX addition. The abundance of these proteins was also compared between the CHX-treated *artΔ* and WT strains to determine whether the α-arrestin gene deletion disrupts the effect of CHX. A protein abundance ratio between two conditions corresponds to the fold change of its proportion in the total amount of PM proteins between the two conditions because the same amount of protein of each condition was analyzed. The logarithmic mean of the replicates of each protein ratio and the corresponding pooled *p*-value were computed using R software. Briefly, the weighted average of *p*-value-corresponding z-scores were calculated (considering a normal distribution) using the number of peptides used for protein quantification as weights [66,67]. The average z-scores were converted to their original corresponding probability and corrected using the Bonferroni method. The significance of each ratio is taken into account during hypothesis testing (H_0_: ratio ≠ 1), which is associated to a *p*-value. Red and blue ratios are significantly different from 1 (i.e., characterized by a *p*-value < 0.05). The false discovery rate (FDR) of the mass spectrometry analysis is lower than 1%, and all the proteins (Tables 1 and 2) were identified with an Unused ProtScore of at least 2 in the ProteinPilot software.

### Total protein extraction

Yeast cells were grown at 28 °C to early exponential phase in 5 or 50 ml of thiamine-free selective medium. Endocytosis was triggered by addition of thiamine, oxythiamine (Sigma-Aldrich) (final concentration of 100 μM), or CHX (final concentration 50 μg/ml). After 2 h of incubation, cells were harvested by centrifugation at 3,000*g* for 5 min, washed with cold distilled water, and resuspended in 200 μl lysis buffer (250 mM sorbitol, 50 mM imidazole, 1 mM MgCl_2_.6H_2_O [pH 7.5]) containing phenylmethylsulfonyl fluoride (PMSF) at a final concentration of 1 mM, 0.1% of a cocktail of protease inhibitors (PICs) (Roche), and N-ethylmaleimide (NEM) at a final concentration of 10 mM. Cells were lysed with glass beads. Cell debris and unbroken cells were eliminated by centrifugation at 3,000*g* for 20 s. The supernatant was mixed with an equal volume of sample buffer (100 mM Tris HCl [pH 6.8], 4 mM EDTA, 4% SDS, 20% glycerol, and 0.002% bromophenol blue) containing 1% dithiothreitol (DTT) for immunoblotting. The protein concentration was determined using a bicinchoninic acid (BCA) assay [68].

### Immunoprecipitation of Thi7-GFP

Yeast cells were grown at 28 °C to an OD_600nm_ of 1 in 500 ml of thiamine-free selective medium. Total protein extraction was performed as described above but using a Triton X-100 containing lysis buffer (1% Triton X-100, 50 mM Tris, 150 mM NaCl, 1 mM PMSF, 0.1% PIC, 10 mM NEM [pH 7.4]). Protein concentration of the extracts was quantified by a BCA assay. Total protein extracts were incubated with 5 mM n-Dodecyl β-D-maltoside (DDM) for 3 h at 4 °C. After centrifugation at 100,000*g* for 1 h, the supernatant was recovered, and immunoprecipitation was then carried out according to GFP-Trap manufacturer’s protocol (Chromotek, Planegg-Martinsried, Germany). Equilibration and washing buffers have the same composition as the lysis buffer, supplemented with 5 mM and 0.5 mM DDM, respectively. A volume of 25 μl of GFP-Trap magnetic agarose beads were mixed with 500 μl of equilibration buffer and separated from the supernatant, which was discarded. The protein lysate was added to the equilibrated beads and incubated for 2 h at 4 °C. Beads were harvested and the supernatant (flow-through) kept for immunoblotting. Beads were washed five times in washing buffer. Elution was carried out using a 0.2 M glycine solution (pH 2.5), supplemented with 1 mM PMSF, 0.1% PIC, 10 mM NEM, and 0.5 mM DDM. The eluate was immediately neutralized with 5 μl of 1 M Tris-containing neutralization solution (pH 10.4), supplemented with 1 mM PMSF, 0.1% PIC, 10 mM NEM, and 0.5 mM DDM.

### Immunoblotting and antibodies

Proteins were separated by SDS-PAGE, transferred to a polyvinylidene fluoride membrane (Millipore) using the Trans-Blot Turbo Transfer System (Bio-Rad), and probed with a mouse monoclonal anti-GFP (Roche), rabbit polyclonal anti-GFP (Abcam), rat anti-HA (Roche), mouse anti-ubiquitin (Thermo Fisher Scientific), or rabbit monoclonal anti-Pma1 [64], followed by incubation with horseradish peroxidase–coupled anti-rat, anti-rabbit, or anti-mouse IgG antibodies (dilution 1:10,000) and chemiluminescence detection (Roche Diagnostics). Chemiluminescence was captured using an Amersham Imager 600 (GE Healthcare). Signals were quantified using Fiji software [69].

### Growth tests on oxythiamine-containing medium

Transformed *thi7Δnrt1Δthi72Δ* strains were grown overnight in thiamine-free selective (SC-U-B1) liquid medium. Cultures were adjusted to an OD_600nm_ of 0.4, and four 10-fold serial dilutions were spotted onto SC-U-B1 solid medium containing oxythiamine at different concentrations.

### Growth tests on thiamine-containing medium

Transformed *thi7Δnrt1Δthi72Δthi4Δ* strains were grown overnight in thiamine-free liquid medium supplemented with 10 nM of thiamine. Cultures were adjusted to an OD_600nm_ of 0.4 and resuspended in thiamine-free selective (SC-U-B1) liquid medium. Then, 10-fold serial dilutions were spotted onto SC-U-B1 solid medium containing thiamine at different concentrations.

### Fluorescence microscopy

Yeast cells were grown at 28 °C up to exponential phase in thiamine-free selective medium. The addition of thiamine, oxythiamine (final concentration of 100 μM), CHX (final concentration of 50 μg/ml), or rapamycin (100 or 200 ng/ml) triggered endocytosis. After 0 and 180 min, 5 μl cellular suspension was dropped onto a microscope slide covered by agarose 1.5% (w/v). The fluorescence analysis was performed with a Leica DMR microscope equipped with a 100× oil-immersion objective and a Hamatsu Orca- ER digital camera. Cells were observed at room temperature using appropriate filter sets. For FM4–64 vacuolar staining, cells were incubated at 28 °C for 45 min with 8 μM FM4–64 dye (Thermo Scientific), washed twice with thiamine-free selective medium, and incubated for endocytosis experiments. For each sample, a corresponding Nomarski picture was taken. Images were processed with HOKAWA! Imaging pf07 2.1 software. In some experiments, fluorescence quantification was performed using Fiji software [69].

### Fluorescence quantification

The fluorescence intensity of Thi7-GFP and mutants was quantified using the Fiji platform from ImageJ. Two ellipses were manually drawn, with the first one surrounding the cell and the second one delimiting the inside, excluding the PM. The median with 95% confidence interval of the ratio of internal-over-PM intensity for each condition is presented. We measured internal instead of vacuolar fluorescence intensities to consider internalized transporters localizing in nonvacuolar intracellular compartments. Wilcoxon tests were used to assess the significance of internal-over-PM fluorescence ratio. In case of multiple tests with a same set of data, *p*-values were adjusted using the Holm method.

### Structural modeling of Thi7 and docking of thiamine

A BLAST search performed on the Protein Data Bank (PDB) sequences identified the benzyl-hydantoin transporter Mhp1 sequence as similar to Thi7. An alignment between Thi7 and Mhp1 sequences was performed with PROMALS3D, also using information from the crystal structure of Mhp1 [70]. Five 3D models of Thi7 were built by comparative modeling with Modeller9v11 [71] using the OF open, occluded (substrate-bound), and IF open crystal structures of Mhp1 (PDB codes 2JLN, 4D1B, and 2X79) as templates [40–42] based on the PROMALS3D alignment. In each case, the modelization of Thi7 was performed from residue A22 to residue Y532. Docking of thiamine (PubChem code 1130) was performed in the occluded models of Thi7 with Glide [72]. Thiamine input file was prepared with the LigPrep module of Schrödinger. Prior to docking, transporter preparation was carried out using the Protein Preparation Wizard protocol (Schrödinger software). A grid receptor box with the dimensions adapted to the benzyl-hydantoin was generated. Hydroxyls of serine, threonine, or tyrosine and thiol of cysteine residues in the binding site were specified as rotatable groups. The Glide SP function was chosen to score the docking poses of the ligand. The remaining parameters were set at their default values. The Schrödinger maestro graphics interface was used to analyze all poses and models.

## Supporting information

S1 Fig Amino- and carboxy-terminal GFP fusions of THI7, NRT1, and THI72 are functional. (A) Phenotypic growth test of the *thi7Δnrt1Δthi72Δ* strain expressing native N- and C-terminal GFP-fused version of THI7, NRT1, and THI72 transporters on thiamine-free medium and thiamine-free selective (SC-U-B1) medium supplemented with oxythiamine (final concentration: 3 μM). (B) Phenotypic growth test of the *thi7Δnrt1Δthi72Δthi4Δ* strain expressing native and C-terminal GFP-fused version of THI7, NRT1, and THI72 transporters on thiamine-free selective (SC-U-B1) medium or supplemented with thiamine. Representative of 2 independent experiments. e.v., empty vector; Nrt1, nicotinamide riboside transporter 1; GFP, green fluorescent protein.
(TIF)

S2 Fig Thiamine-induced endocytosis of Thi7-GFP, Nrt1-GFP, and Thi72-GFP in all remaining *artΔ* strains. Localization of Thi7-GFP, Nrt1-GFP, or Thi72-GFP in all *artΔ* strains (excepted *art2Δ* and *art9Δ* shown in Figs 1 and 2) after thiamine addition (final concentration: 100 μM) into culture grown in thiamine-free medium. Scale bar represents 5 μM. GFP, green fluorescent protein; Nrt1, nicotinamide riboside transporter 1.
(TIF)

S3 Fig Addition of oxythiamine induces Thi7 endocytosis. Localization of Thi7-GFP in a WT strain after oxythiamine addition (final concentration: 100 μM) into culture grown in thiamine-free selective medium. Scale bar represents 5 μm. GFP, green fluorescent protein; WT, wild type.
(TIF)

S4 Fig Single-point Thi7 mutants display small variations in protein cellular abundance. The *thi7Δnrt1Δthi72Δ* strain expressing single-point *THI7-GFP* mutants, wild-type *THI7-GFP*, or e.v. were grown in thiamine-free selective medium to early-log phase before being harvested. Extracts were immunoblotted with anti-GFP and anti-Pma1 as a loading control. Values below Thi7-GFP bands are quantification of the relative band intensity normalized by the intensity of Pma1-corresponding band (mean from 3 independent experiments). e.v., empty vector; GFP, green fluorescent protein; Pma1, plasma membrane ATPase 1.
(TIF)

S5 Fig Thi7^T80K^ and Thi7^S338L^ are retained within the endoplasmic reticulum in 25% of the cells. Localization of Thi7-GFP, Thi7^T80K^-GFP, and Thi7^S338L^-GFP in a *thi7Δnrt1Δthi72Δ* strain into culture grown in thiamine-free selective medium. Scale bar represents 5 μm. GFP, green fluorescent protein.
(TIF)

S6 Fig Phenotypic growth test of a *thi7Δnrt1Δthi72Δthi4Δ* strain expressing *THI7*^*M399R*^*-GFP* on thiamine-supplemented medium. Phenotypic growth test of a *thi7Δnrt1Δthi72Δthi4Δ* strain expressing an e.v. or *THI7*^*M399R*^*-GFP* on thiamine-free selective medium (SC-U-B1) or supplemented with thiamine. Representative of 4 independent experiments. e.v., empty vector; GFP, green fluorescent protein.
(TIF)

S7 Fig 3D models of Thi7 in OF (green), occluded (yellow), and IF (red) conformations with docked thiamine. (Left panel) Thi7, in an OF open conformation, clearly displays a cavity for the substrate to enter and bind. (Second and third panels) Thi7, in an occluded state, exhibits no cavity from both the top and bottom view. (Right panel) Thi7, in an IF open conformation, displays a cavity from which thiamine is released. 3D, three-dimensional; IF, inward-facing; OF, outward-facing.
(TIF)

S8 Fig HA-Npr1 does not undergo phosphorylation upon thiamine addition at early time points. A WT strain expressing *HA-NPR1* and complemented with the pFL36 plasmid was grown up to early log-phase in ammonium-containing thiamine-free complete medium (“Am + a.a.–Thiamine”) and incubated for 5, 15, 30, and 180 min with thiamine (100 μM) before being harvested. Cell extracts were immunoblotted with anti-HA and anti-Pma1 antibodies. HA, hemagglutinin; Pma1, plasma membrane ATPase 1; WT, wild type.
(TIF)

S1 Table List of identified plasma membrane proteins in the proteomic screening. (DOCX)

S2 Table Minimum and maximum values of ratios of identified plasma membrane proteins in the proteomic screening. (XLSX)

S3 Table Strains used in this study. (DOCX)

S4 Table Plasmids used in this study. (DOCX)

S1 Data Numerical data of CHX-induced and thiamine-induced Thi7 endocytosis. CHX, cycloheximide.
(XLSX)

S2 Data Numerical data of thiamine-induced Nrt1 and Thi72 endocytosis. Nrt1, nicotinamide riboside transporter 1.
(XLSX)

S3 Data Numerical data of thiamine-induced endocytosis of transport-defective mutants. (XLSX)

S4 Data Numerical data of endocytosis of Thi7^M399R^-GFP, Thi7^N350K^-GFP, and Thi7-GFP when coexpressed with Thi7^6KR^. GFP, green fluorescent protein.
(XLSX)

S5 Data Numerical data of endocytosis of Thi7^D85G^-GFP and Thi7^P291Q^-GFP when coexpressed with Thi7^6KR^. GFP, green fluorescent protein.
(XLSX)

S6 Data Numerical data of Npr1 analysis and rapamycin-induced Thi7 endocytosis. (XLSX)

## Abbreviations

3D: three-dimensional
ABC: ATP-binding cassette
CHX: cycloheximide
e.v.: empty vector
ER: endoplasmic reticulum
GFP: green fluorescent protein
GPI: glycosylphosphatidylinositol
GTP: guanosine triphosphate
HA: hemagglutinin
HECT: homologous to E6 associated protein carboxyl terminus
Hxt: hexose transporter
IF: inward-facing
KR: lysine-to-arginine
LID: loop-interaction domain
Lyp1: lysine-specific permease 1
MCC: membrane compartment containing Can1
Mhp1: membrane hydantoin transport protein 1
MVB: multivesicular body
NCS1: nucleobase-cation-symport-1
Nrt1: nicotinamide riboside transporter 1
ns: nonsignificant
OF: outward-facing
PM: plasma membrane
Pma1: plasma membrane ATPase 1
Ptr2: peptide transporter 2
TM7: seventh transmembrane span
TORC1: target of rapamycin complex 1
TPP: thiamine pyrophosphate
WT: wild type
