# Supplementary figures and images for: Yeast α-arrestin Art2 is the key regulator of ubiquitylation-dependent endocytosis of plasma membrane vitamin B1 transporters
Source: PLoS Biol. 2019 Oct 28;17(10):e3000512. doi: 10.1371/journal.pbio.3000512 (PMC6837554; doi:10.1371/journal.pbio.3000512)

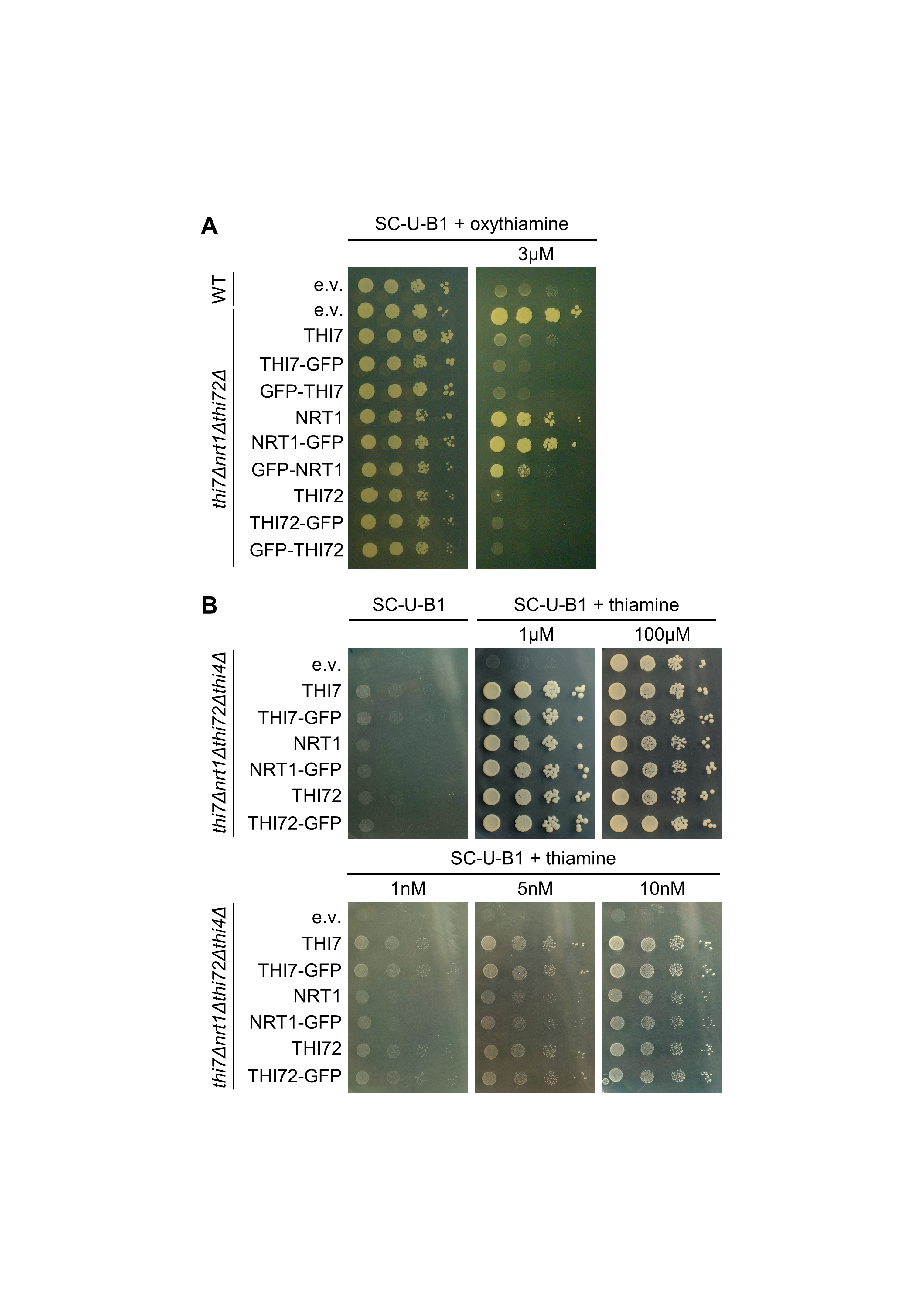

Supplement: S1 Fig — (A) Phenotypic growth test of the thi7Δnrt1Δthi72Δ strain expressing native N- and C-terminal GFP-fused version of THI7, NRT1, and THI72 transporters on thiamine-free medium and thiamine-free selective (SC-U-B1) medium supplemented with oxythiamine (final concentration: 3 μM). (B) Phenotypic growth test of the thi7Δnrt1Δthi72Δthi4Δ strain expressing native and C-terminal GFP-fused version of THI7, NRT1, and THI72 transporters on thiamine-free selective (SC-U-B1) medium or supplemented with thiamine. Representative of 2 independent experiments. e.v., empty vector; Nrt1, nicotinamide riboside transporter 1; GFP, green fluorescent protein. (TIF) [file pbio.3000512.s001.tif]

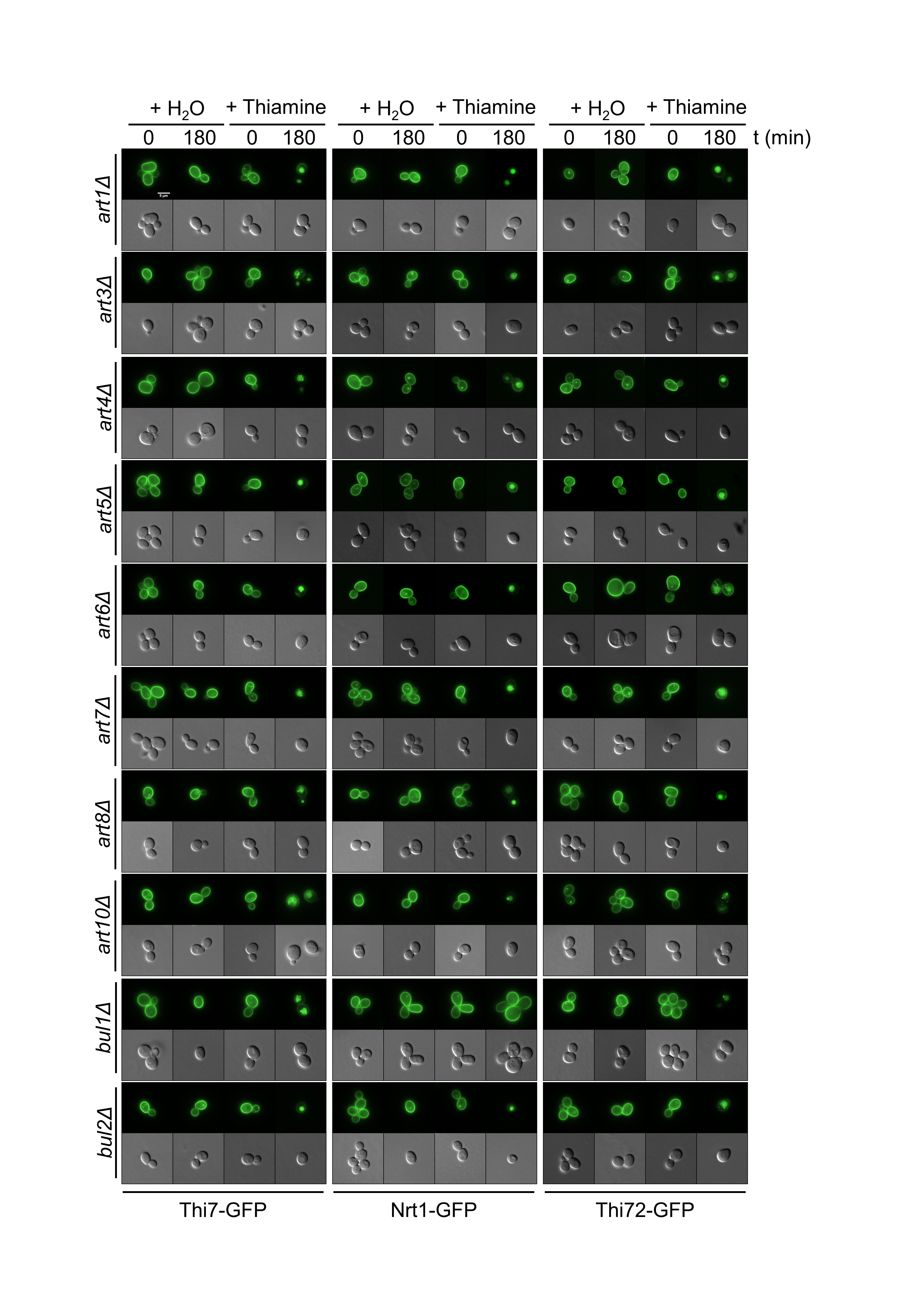

Supplement: S2 Fig — Localization of Thi7-GFP, Nrt1-GFP, or Thi72-GFP in all artΔ strains (excepted art2Δ and art9Δ shown in Figs 1 and 2) after thiamine addition (final concentration: 100 μM) into culture grown in thiamine-free medium. Scale bar represents 5 μM. GFP, green fluorescent protein; Nrt1, nicotinamide riboside transporter 1. (TIF) [file pbio.3000512.s002.tif]

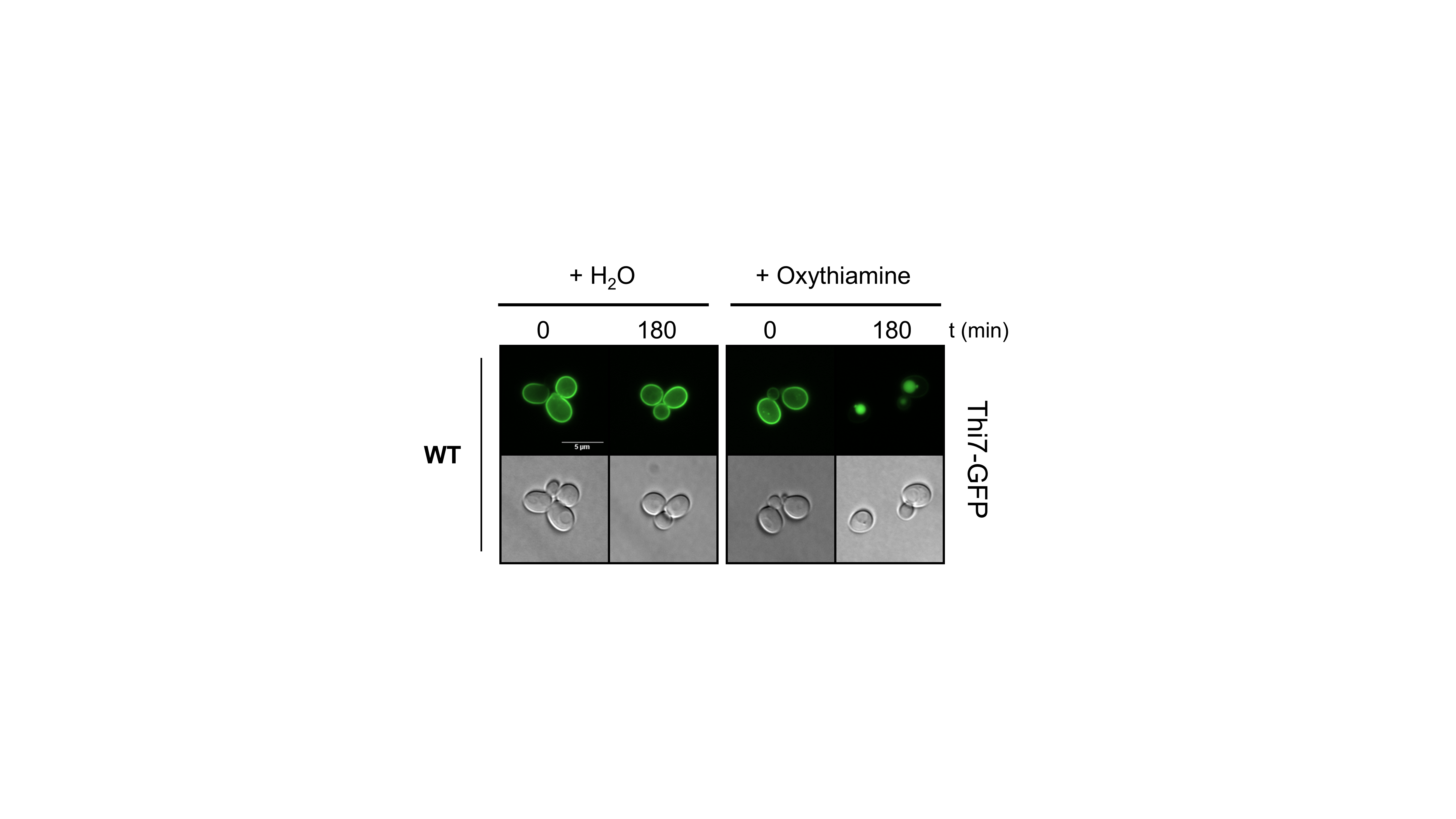

Supplement: S3 Fig — Localization of Thi7-GFP in a WT strain after oxythiamine addition (final concentration: 100 μM) into culture grown in thiamine-free selective medium. Scale bar represents 5 μm. GFP, green fluorescent protein; WT, wild type. (TIF) [file pbio.3000512.s003.tif]

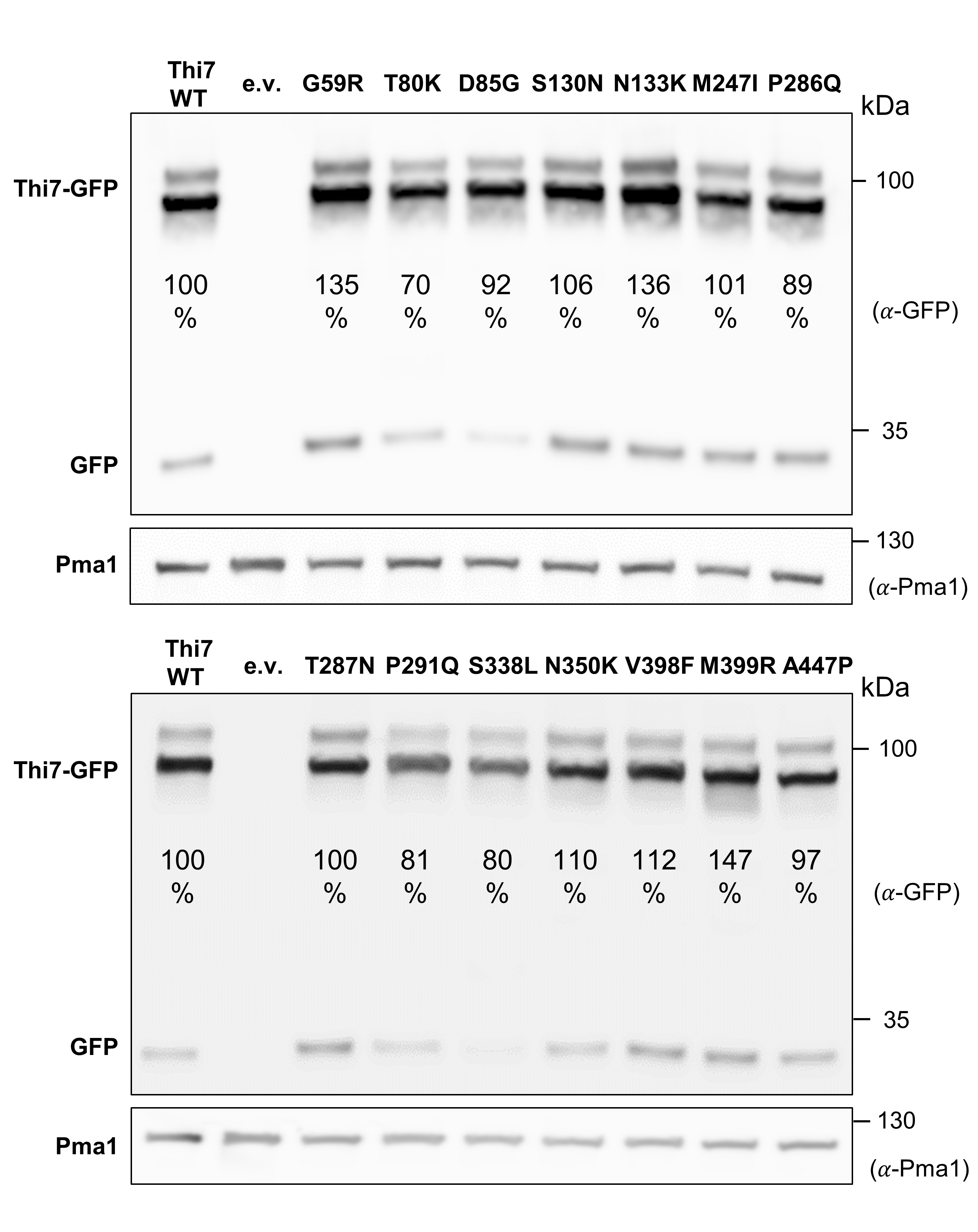

Supplement: S4 Fig — The thi7Δnrt1Δthi72Δ strain expressing single-point THI7-GFP mutants, wild-type THI7-GFP, or e.v. were grown in thiamine-free selective medium to early-log phase before being harvested. Extracts were immunoblotted with anti-GFP and anti-Pma1 as a loading control. Values below Thi7-GFP bands are quantification of the relative band intensity normalized by the intensity of Pma1-corresponding band (mean from 3 independent experiments). e.v., empty vector; GFP, green fluorescent protein; Pma1, plasma membrane ATPase 1. (TIF) [file pbio.3000512.s004.tif]

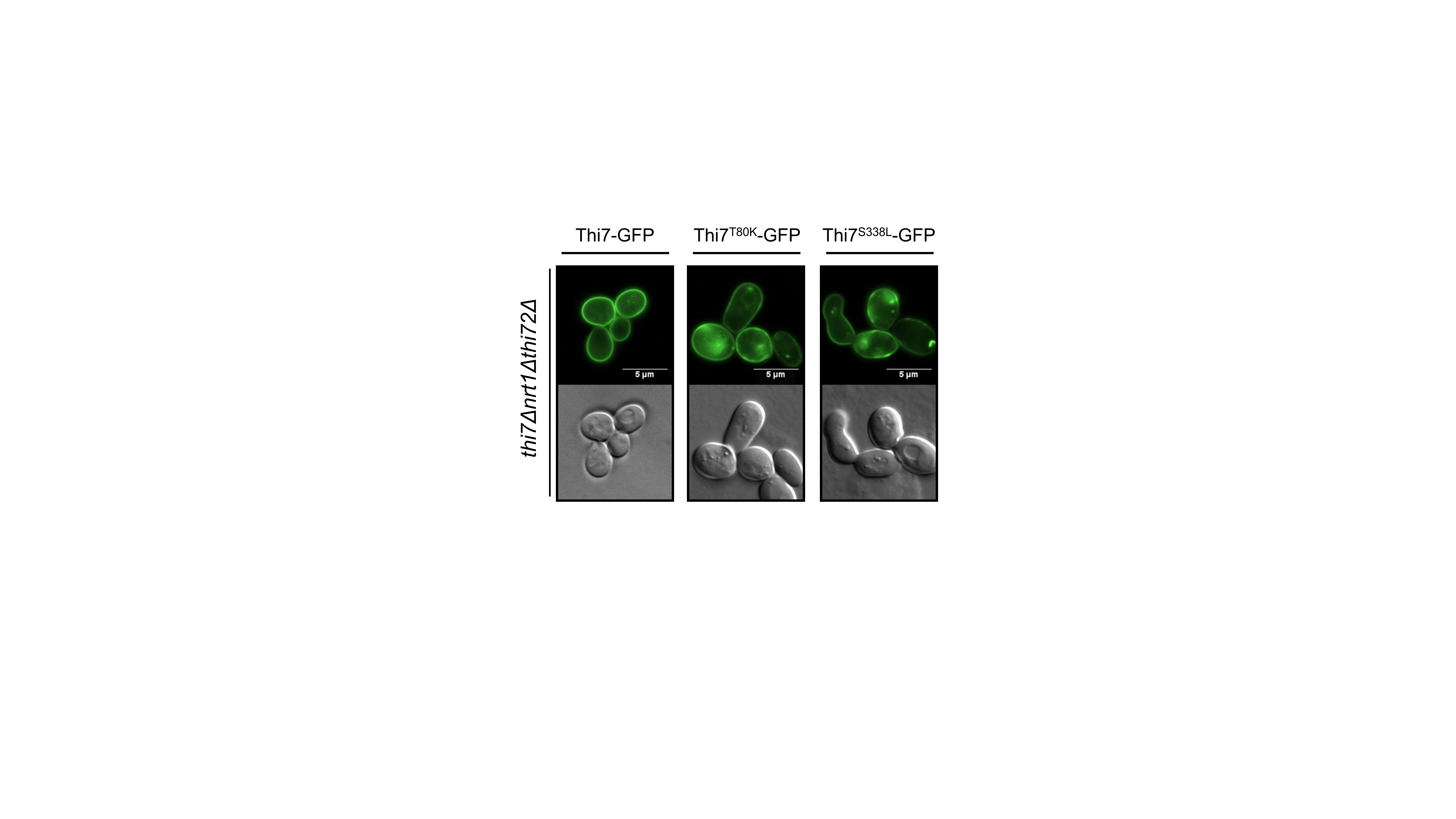

Supplement: S5 Fig — Localization of Thi7-GFP, Thi7T80K-GFP, and Thi7S338L-GFP in a thi7Δnrt1Δthi72Δ strain into culture grown in thiamine-free selective medium. Scale bar represents 5 μm. GFP, green fluorescent protein. (TIF) [file pbio.3000512.s005.tif]

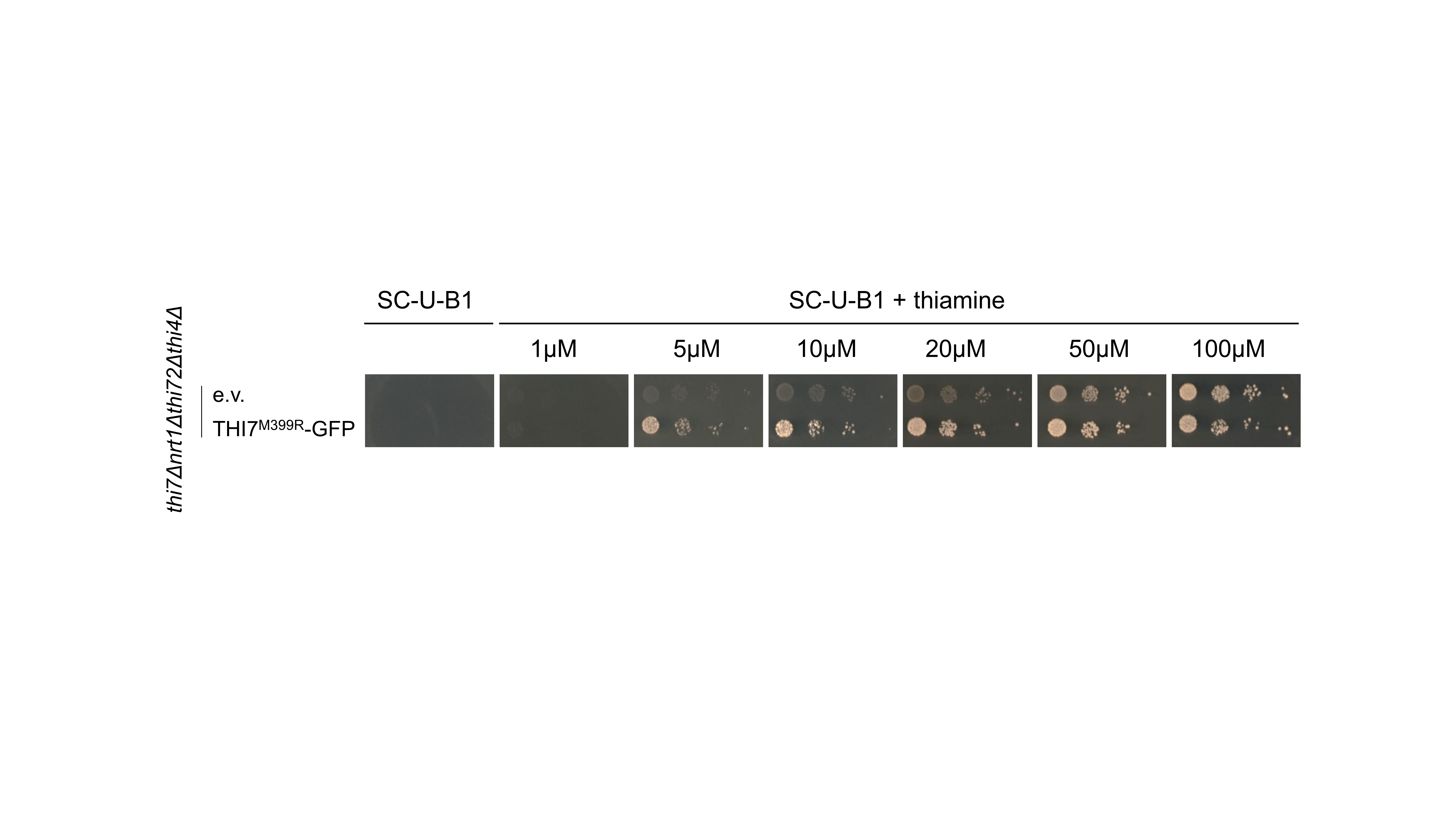

Supplement: S6 Fig — Phenotypic growth test of a thi7Δnrt1Δthi72Δthi4Δ strain expressing an e.v. or THI7M399R-GFP on thiamine-free selective medium (SC-U-B1) or supplemented with thiamine. Representative of 4 independent experiments. e.v., empty vector; GFP, green fluorescent protein. (TIF) [file pbio.3000512.s006.tif]

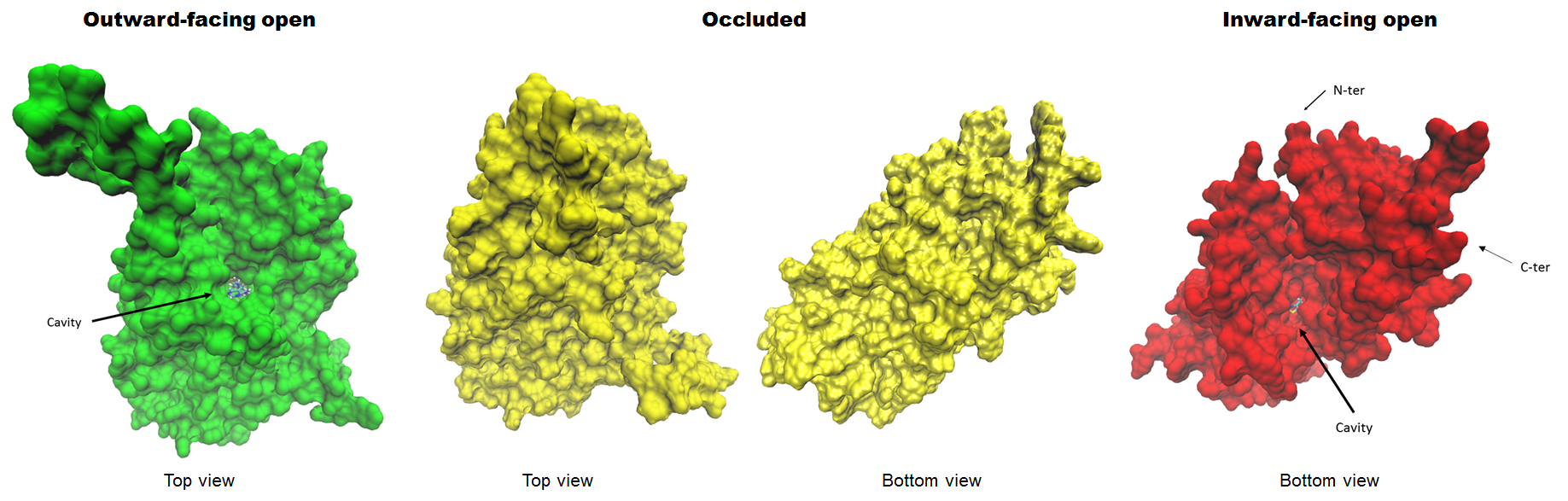

Supplement: S7 Fig — (Left panel) Thi7, in an OF open conformation, clearly displays a cavity for the substrate to enter and bind. (Second and third panels) Thi7, in an occluded state, exhibits no cavity from both the top and bottom view. (Right panel) Thi7, in an IF open conformation, displays a cavity from which thiamine is released. 3D, three-dimensional; IF, inward-facing; OF, outward-facing. (TIF) [file pbio.3000512.s007.tif]

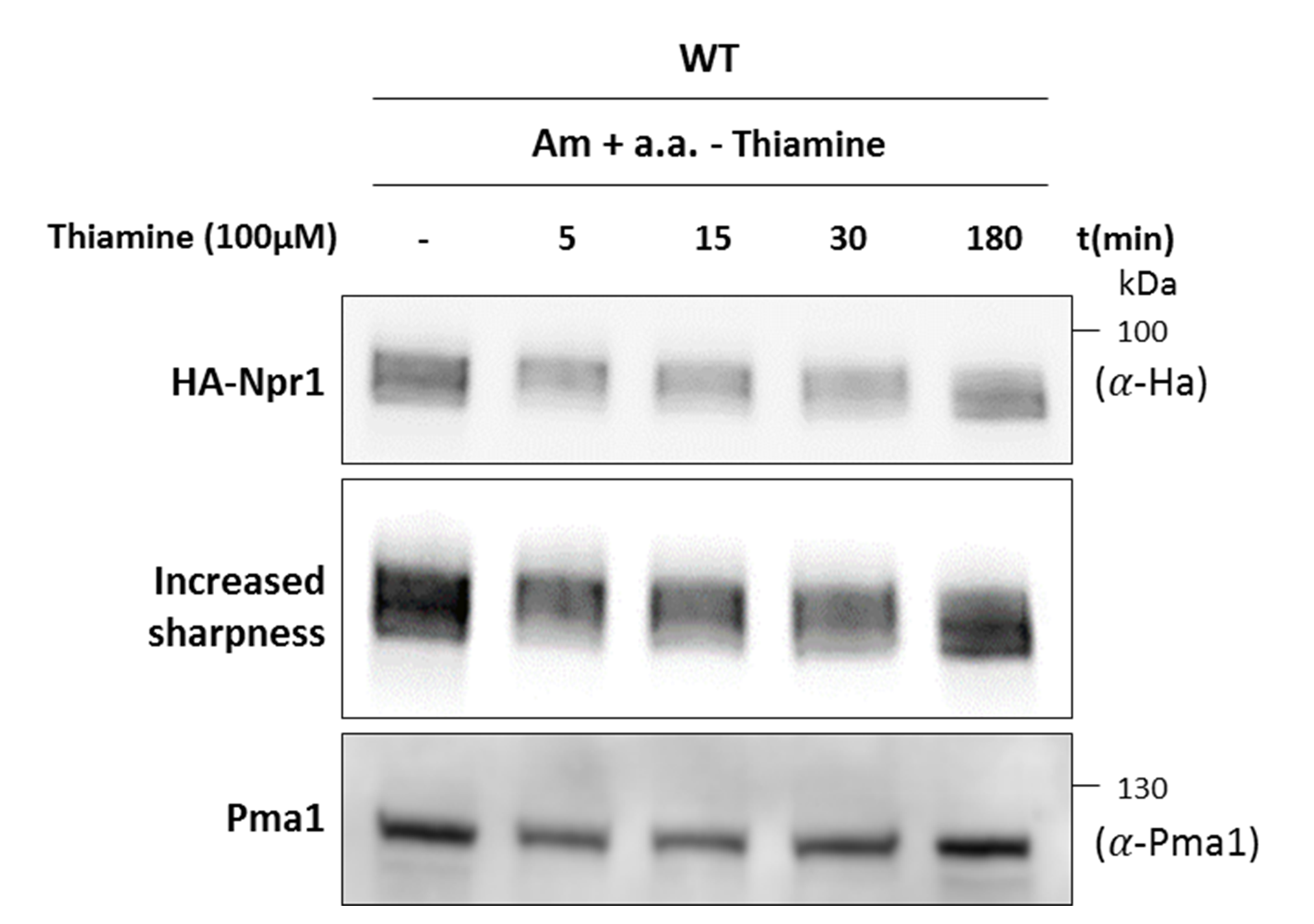

Supplement: S8 Fig — A WT strain expressing HA-NPR1 and complemented with the pFL36 plasmid was grown up to early log-phase in ammonium-containing thiamine-free complete medium (“Am + a.a.–Thiamine”) and incubated for 5, 15, 30, and 180 min with thiamine (100 μM) before being harvested. Cell extracts were immunoblotted with anti-HA and anti-Pma1 antibodies. HA, hemagglutinin; Pma1, plasma membrane ATPase 1; WT, wild type. (TIF) [file pbio.3000512.s008.tif]
