# Supplementary material for: Yeast α-arrestin Art2 is the key regulator of ubiquitylation-dependent endocytosis of plasma membrane vitamin B1 transporters
Source: PLoS Biol. 2019 Oct 28;17(10):e3000512. doi: 10.1371/journal.pbio.3000512 (PMC6837554; doi:10.1371/journal.pbio.3000512)
Supplement: S1 Table — (DOCX) [file pbio.3000512.s009.docx]

**Table S1.** List of identified plasma membrane proteins.

| **Accession** | **Systematic name** | **Alias** | **Description** |
| --- | --- | --- | --- |
| P01119 | YOR101W | RAS1 | GTPase involved in adenylate cyclase activation |
| P01120 | YNL098C | RAS2 | GTPase involved in adenylate cyclase activation |
| P04817 | YEL063C | CAN1 | Arginine permease |
| P05030 | YGL008C | PMA1 | P2-type H^+^-ATPase |
| P06780 | YPR165W | RHO1 | Small GTPase of the Rho/Rac subfamily of Ras-like proteins |
| P06781 | YNL090W | RHO2 | Small GTPase of the Rho/Rac subfamily of Ras-like proteins |
| P08539 | YHR005C | GPA1 | GTP-binding α-subunit of the heterotrimeric G protein |
| P10823 | YER020W | GPA2 | GTP-binding α-subunit of the heterotrimeric G protein |
| P13856 | YGR152C | RSR1 | GTP-binding protein of the Ras superfamily |
| P18852 | YJR086W | STE18 | G protein γ-subunit |
| P19073 | YLR229C | CDC42 | Small Rho-like GTPase |
| P22146 | YMR307W | GAS1 | Beta-1,3-glucanosyltransferase |
| P23291 | YHR135C | YCK1 | Palmitoylated plasma membrane-bound casein kinase I (CK1) isoform |
| P23292 | YNL154C | YCK2 | Palmitoylated plasma membrane-bound casein kinase I (CK1) isoform |
| P23900 | YLL043W | FPS1 | Aquaglyceroporin |
| P25618 | YCR017C | CWH43 | GPI lipid remodelase |
| P30605 | YDR497C | ITR1 | Myo-inositol transporter |
| P30606 | YOL103W | ITR2 | Myo-inositol transporter |
| P32329 | YLR120C | YPS1 | Aspartic protease |
| P32465 | YHR094C | HXT1 | Low-affinity glucose transporter |
| P32466 | YDR345C | HXT3 | Low affinity glucose transporter |
| P32467 | YHR092C | HXT4 | High-affinity glucose transporter |
| P32568 | YDR011W | SNQ2 | ATP-binding cassette (ABC) transporter |
| P32660 | YER166W | DNF1 | Aminophospholipid translocase (flippase) |
| P32791 | YLR214W | FRE1 | Ferric and cupric reductase |
| P32867 | YPL232W | SSO1 | Plasma membrane t-SNARE |
| P32901 | YKR093W | PTR2 | Integral membrane peptide transporter |
| P33302 | YOR153W | PDR5 | Plasma membrane ATP-binding cassette (ABC) transporter |
| P35735 | YKL051W | SFK1 | Plasma membrane protein putatively acting on PI4P level |
| P36027 | YLR332W | MID2 | Protein involved in cell wall integrity signaling |
| P36091 | YKL046C | DCW1 | Putative mannosidase |
| P38079 | YBR054W | YRO2 | Protein with a putative role in response to acid stress |
| P38085 | YBR069C | TAT1 | Amino acid (valine, leucine, isoleucine, and tyrosine) transporter |
| P38227 | YBR043C | QDR3 | Transporter of the Drug:H+ antiporter family |
| P38248 | YBR078W | ECM33 | GPI-anchored protein of unknown function |
| P38250 | YBR086C | IST2 | Cortical ER protein involved in ER-plasma membrane tethering |
| P38631 | YLR342W | FKS1 | Catalytic subunit of the 1,3-beta-D-glucan synthase |
| P38993 | YMR058W | FET3 | Ferro-O_2_-oxidoreductase |
| P39004 | YDR342C | HXT7 | High-affinity glucose transporter |
| P39105 | YMR008C | PLB1 | Phospholipase B (lysophospholipase) |
| P39926 | YMR183C | SSO2 | Plasma membrane t-SNARE |
| P39928 | YIL147C | SLN1 | Transmembrane histidine phosphotransfer kinase and osmosensor |
| P40073 | YER118C | SHO1 | Osmosensor for filamentous growth and HOG pathways |
| P40088 | YER145C | FTR1 | High-affinity iron permease |
| P40474 | YIL121W | QDR2 | Transporter of the Drug/H^+^ antiporter family |
| P40485 | YIL105C | SLM1 | Phosphoinositide binding protein |
| P41930 | YPL092W | SSU1 | Sulfite pump |
| P42838 | YNL323W | LEM3 | Protein of unknown function |
| P46950 | YGR197C | SNG1 | Protein involved in resistance to nitrosoguanidine and 6-azauracil |
| P46951 | YGR198W | YPP1 | Cargo-transport protein involved in endocytosis |
| P48231 | YNL087W | TCB2 | ER protein involved in ER-plasma membrane tethering |
| P49573 | YPR124W | CTR1 | High-affinity copper transporter of plasma membrane |
| P52911 | YDR261C | EXG2 | Exo-1,3-beta-glucanase |
| P53049 | YGR281W | YOR1 | Plasma membrane ATP-binding cassette (ABC) transporter |
| P53154 | YGL084C | GUP1 | Protein involved in remodeling GPI anchors |
| P53283 | YGR138C | TPO2 | Polyamine transporter |
| P53388 | YPL265W | DIP5 | Dicarboxylic amino acid permease |
| P53390 | YPR138C | MEP3 | Ammonium permease |
| P53879 | YNL180C | RHO5 | Small GTPase of the Rho/Rac family of Ras-like proteins |
| P54003 | YML052W | SUR7 | Transmembrane component of eisosomes |
| Q00245 | YIL118W | RHO3 | Small GTPase of the Rho/Rac family of Ras-like proteins |
| Q01896 | YDR039C | ENA2 | P-type Na^+^-ATPase exporter |
| Q02785 | YPL058C | PDR12 | Plasma membrane ATP-binding cassette (ABC) transporter |
| Q03640 | YML072C | TCB3 | Cortical ER protein involved in ER-plasma membrane tethering |
| Q04182 | YDR406W | PDR15 | Plasma membrane ATP binding cassette (ABC) transporter |
| Q05050 | YMR031C | EIS1 | Component of the eisosomes |
| Q05998 | YLR237W | THI7 | High-affinity thiamine transporter |
| Q06170 | YLR326W |  | Putative protein of unknown function |
| Q06451 | YPR156C | TPO3 | Polyamine transporter |
| Q06689 | YLR413W | INA1 | Protein of unknown function |
| Q06991 | YLR414C | PUN1 | Protein involved in cell wall integrity |
| Q07800 | YLL010C | PSR1 | Plasma membrane-associated protein phosphatase |
| Q07824 | YLL028W | TPO1 | Polyamine transporter |
| Q07904 | YLR004C | THI73 | Putative plasma membrane permease |
| Q07950 | YLR020C | YEH2 | Steryl ester hydrolase |
| Q08245 | YOL109W | ZEO1 | Peripheral protein involved in cell wall integrity |
| Q08269 | YOL130W | ALR1 | Mg^2+^ transporter |
| Q08417 | YOR049C | RSB1 | Putative sphingoid long-chain base (LCB) efflux transporter |
| Q08760 | YOR301W | RAX1 | Protein involved in bud site selection during bipolar budding |
| Q08986 | YPL274W | SAM3 | High-affinity S-adenosylmethionine permease |
| Q12117 | YDR033W | MRH1 | Protein of unknown function |
| Q12207 | YPR149W | NCE102 | Transmembrane component of eisosomes |
| Q12246 | YOR171C | LCB4 | Sphingoid long-chain base kinase |
| Q12256 | YOR273C | TPO4 | Polyamine transporter |
| Q12359 | YDR384C | ATO3 | Putative ammonium transporter |
| Q12361 | YDL035C | GPR1 | G protein-coupled receptor |
| Q12412 | YOR161C | PNS1 | Protein of unknown function |
| Q12465 | YLR084C | RAX2 | N-glycosylated protein |
| Q12466 | YOR086C | TCB1 | Lipid-binding ER protein involved in ER-plasma membrane tethering |
| Q12675 | YDR093W | DNF2 | Aminophospholipid translocase (flippase) |
| Q12746 | YML125C | PGA3 | Putative cytochrome b5 reductase |
| Q3E756 | YBL029C-A |  | Protein of unknown function |
| Q99271 | YLR138W | NHA1 | Na+/H+ antiporter |
