# Supplementary material for: Yeast α-arrestin Art2 is the key regulator of ubiquitylation-dependent endocytosis of plasma membrane vitamin B1 transporters
Source: PLoS Biol. 2019 Oct 28;17(10):e3000512. doi: 10.1371/journal.pbio.3000512 (PMC6837554; doi:10.1371/journal.pbio.3000512)
Supplement: S3 Table — (DOCX) [file pbio.3000512.s011.docx]

**Table S3.** Strains used in this study.

| **Strain (alias)** | **Genotype** | **Source** |
| --- | --- | --- |
| Wild-Type (WT) | BY4742; Mat α; *his3Δ1*; *leu2Δ0*; *lys2Δ0*; *ura3Δ0* | EUROSCARF |
| *art1∆* | BY4742; Mat α; *his3Δ1*; *leu2Δ0*; *lys2Δ0*; *ura3Δ0*; *YOR322c*::kanMX4 | EUROSCARF |
| *art2∆* | BY4742; Mat α; *his3Δ1*; *leu2Δ0*; *lys2Δ0*; *ura3Δ0*; *YBL101c*::kanMX4 | EUROSCARF |
| *art2∆* | BY4741; Mat a; *his3∆1*; *leu2∆0*; *met15∆0*; *ura3∆0*; *YBL101c*::kanMX4 | EUROSCARF |
| *art3∆* | BY4742; Mat α; *his3∆1*; *leu2∆0*; *lys2∆0*; *ura3∆0*; *YJL084c*::kanMX4 | EUROSCARF |
| *art4∆* | BY4742; Mat α; *his3∆1*; *leu2∆0*; *lys2∆0*; *ura3∆0*; *YOR018w*::kanMX4 | EUROSCARF |
| *art5∆* | BY4742; Mat α; *his3∆1*; *leu2∆0*; *lys2∆0*; *ura3∆0*; *YGR068c*::kanMX4 | EUROSCARF |
| *art6∆* | BY4742; Mat α; *his3∆1*; *leu2∆0*; *lys2∆0*; *ura3∆0*; *YKR021w*::kanMX4 | EUROSCARF |
| *art7∆* | BY4742; Mat α; *his3∆1*; *leu2∆0*; *lys2∆0*; *ura3∆0*; *YFR022w*::kanMX4 | EUROSCARF |
| *art8∆* | BY4742; Mat α; *his3∆1*; *leu2∆0*; *lys2∆0*; *ura3∆0*; *YPR030w*::kanMX4 | EUROSCARF |
| *art9∆* | BY4742; Mat α; *his3∆1*; *leu2∆0*; *met15∆0*; *ura3∆0*; *YGL045w*::kanMX4 | EUROSCARF |
| *art9∆* | BY4741; Mat a; *his3∆1*; *leu2∆0*; *lys2∆0*; *ura3∆0*; *YGL045w*::kanMX4 | EUROSCARF |
| *art10∆* | BY4742; Mat α; *his3∆1*; *leu2∆0*; *lys2∆0*; *ura3∆0*; *YLR392c*::kanMX4 | EUROSCARF |
| *bul1∆* | BY4742; Mat α; *his3∆1*; *leu2∆0*; *lys2∆0*; *ura3∆0*; *YMR275c*::kanMX4 | EUROSCARF |
| *bul2∆* | BY4742; Mat α; *his3∆1*; *leu2∆0*; *lys2∆0*; *ura3∆0*; *YML111w*::kanMX4 | EUROSCARF |
| *end3Δ* | BY4741; Mat α; *his3∆1*; *leu2∆0*; *met15∆0*; *ura3∆0*; *YNL084c*::kanMX4 | EUROSCARF |
| *art2Δart9Δ* | BY4742; Mat α; *his3∆1*; *leu2∆0*; *lys2∆0*; *ura3∆0*; *YBL101c*::kanMX4; *YGL045w*::kanMX4 | This study |
| *end3Δart2Δ* | BY4741; Mat α; *his3∆1*; *leu2∆0*; *met15∆0*; *ura3∆0*; *YNL084c*::kanMX4; *YBL101c*::natNT2 | This study |
| WT *THI7-EYFP* | BY4742; Mat α; *his3Δ1*; *leu2Δ0*; *lys2Δ0*; *ura3Δ0*; *YLR237w-EYFP*::natMX6 | This study |
| *art2∆ THI7-EYFP* | BY4742; Mat α; *his3Δ1*; *leu2Δ0*; *lys2Δ0*; *ura3Δ0*; *YBL101c*::kanMX4; *YLR237w-EYFP*::natMX6 | This study |
| *art9∆ THI7-EYFP* | BY4742; Mat α; *his3∆1*; *leu2∆0*; *met15∆0*; *ura3∆0*; *YGL045w*::kanMX4; *YLR237w-EYFP*::natMX6 | This study |
| *npi1-1 (rsp5)* | BY4741; Mat a; *his3∆1*; *leu2∆0*; *met15∆0*; *ura3∆0*; P*_RSP5_*::kanMX | B. André (ULB, Belgium) |
| *thi7Δnrt1Δthi72Δ* (CVY3) | BY4742; Mat α; *his3∆1*; *leu2∆0*; *lys2∆0*; *ura3∆0*; *YLR237w*::KanMX; *YOR071c*::LEU2; *YOR192c*::LYS2 | J. Stolz (TUM, Germany) |
| *thi7Δnrt1Δthi72Δ*  *thi4Δ* (CVY4) | BY4742; Mat α; *his3∆1*; *leu2∆0*; *lys2∆0*; *ura3∆0*; *YGR144w*::SpHis5; *YLR237w*::KanMX; *YOR071c*::LEU2; *YOR192c*::LYS2 | J. Stolz (TUM, Germany) |
| *sit4Δ* | BY4741; Mat a; *his3Δ1; leu2Δ0; met15Δ0; ura3Δ0;* *YDL047w::*kanMX4 | S. Léon (IJM, France) |
| *npr1Δ* | BY4742; Mat α; *his3Δ1; leu2Δ0; lys2Δ0; ura3Δ0; YNL183c::*kanMX4 | EUROSCARF |
