# Supplementary material for: Yeast α-arrestin Art2 is the key regulator of ubiquitylation-dependent endocytosis of plasma membrane vitamin B1 transporters
Source: PLoS Biol. 2019 Oct 28;17(10):e3000512. doi: 10.1371/journal.pbio.3000512 (PMC6837554; doi:10.1371/journal.pbio.3000512)
Supplement: S4 Table — (DOCX) [file pbio.3000512.s012.docx]

**Table S4.** Plasmids used in this study.

| **Name** | **Plasmid description** | **Source** |
| --- | --- | --- |
| pRS315 | pRS315 CEN *LEU2* | [73] |
| pRS316 | pRS316 CEN *URA3* | [73] |
| pRS416 | pRS416 CEN *URA3* | [73] |
| pRS313 | pRS313 CEN *HIS3* | [73] |
| pFL36-HIS3-LYS2 | pFL36 CEN *LEU2 HIS3 LYS2* | B. André (ULB, Belgium) |
| pFL36-HIS3-MET15 | pFL36 CEN *LEU2 HIS3 MET15* | B. André (ULB, Belgium) |
| 3xHA-ART2 | YCplac111 (*LEU2*); P_ART2_-*3xHA-ART2-*T*_POL4_* | H. Pelham (MRC, UK) |
| YEplac195-THI7-GFP | YEplac195 (2µ *URA3*); P_THI7_-*THI7-yEGFP*-T_ADH1_ | J. Stolz (TUM, Germany) |
| THI7 | pRS416; P_THI7_-*THI7*-T_ADH1_ | This study |
| THI7-GFP | pRS316; P_THI7_-*THI7-yEGFP*-T_ADH1_ | This study |
| GFP-THI7 | pRS316; P_THI7_-yEGFP-*THI7*-T_ADH1_ | This study |
| NRT1 | pRS416; P_THI7_-*NRT1*-T_ADH1_ | This study |
| NRT1-GFP | pRS416; P_THI7_-*NRT1-yEGFP*-T_ADH1_ | This study |
| GFP-NRT1 | pRS416; P_THI7_-yEGFP-*NRT1-*T_ADH1_ | This study |
| THI72 | pRS416; P_THI7_-*THI72*-T_ADH1_ | This study |
| THI72-GFP | pRS416; P_THI7_-*THI72-yEGFP*-T_ADH1_ | This study |
| GFP-THI72 | pRS416; P_THI7_-yEGFP-*THI72-*T_ADH1_ | This study |
| THI7^G59R^-GFP | pRS316; P_THi7_*-THI7^G59R^-yEGFP-*T_ADH1_ | This study |
| THI7^T80K^-GFP | pRS316; P_THi7_*-THI7^T80K^-yEGFP-*T_ADH1_ | This study |
| THI7^D85G^-GFP | pRS316; P_THi7_*-THI7^D85G^-yEGFP-*T_ADH1_ | This study |
| THI7^S130N^-GFP | pRS316; P_THi7_*-THI7^S130N^-yEGFP-*T_ADH1_ | This study |
| THI7^N133K^-GFP | pRS316; P_THi7_*-THI7^N133K^-yEGFP-*T_ADH1_ | This study |
| THI7^M247I^-GFP | pRS316; P_THi7_*-THI7^M247I^-yEGFP-*T_ADH1_ | This study |
| THI7^P286Q^-GFP | pRS316; P_THi7_*-THI7^P286Q^-yEGFP-*T_ADH1_ | This study |
| THI7^T287N^-GFP | pRS316; P_THi7_*-THI7^T287N^-yEGFP-*T_ADH1_ | This study |
| THI7^P291Q^-GFP | pRS316; P_THi7_*-THI7^P291Q^-yEGFP-*T_ADH1_ | This study |
| THI7^S338L^-GFP | pRS316; P_THi7_*-THI7^S338L^-yEGFP-*T_ADH1_ | This study |
| THI7^N350K^-GFP | pRS316; P_THi7_*-THI7^N350K^-yEGFP-*T_ADH1_ | This study |
| THI7^V398F^-GFP | pRS316; P_THi7_*-THI7^V398F^-yEGFP-*T_ADH1_ | This study |
| THI7^M399R^-GFP | pRS316; P_THi7_*-THI7^M399R^-yEGFP-*T_ADH1_ | This study |
| THI7^A447P^-GFP | pRS316; P_THi7_*-THI7^A447P^-yEGFP-*T_ADH1_ | This study |
| THI7^fullKR^-GFP | pRS416; P_THi7_*-THI7^KR^-yEGFP-*T_ADH1_ | This study |
| THI7^NterKR^-GFP | pRS416; P_THi7_*-THI7^K6;19;28;36R^-yEGFP-*T_ADH1_ | This study |
| THI7^CterKR^-GFP | pRS416; P_THi7_*-THI7^K505;509;513;526;527;569;584;588;596R^-yEGFP-*T_ADH1_ | This study |
| THI7^6KR^-GFP | pRS416; P_THi7_*-THI7^K526;527;569;584;588;596R^-yEGFP-*T_ADH1_ | This study |
| THI7-mDsRed | pRS313; P_THi7_*-THI7-mDsRed-*T_ADH1_ | This study |
| THI7^6KR^-mDsRed | pRS313; P_THi7_*-THI7^K526;527;569;584;588;596R^-mDsRed-*T_ADH1_ | This study |
| HA-Npr1 | YEp; HA-NPR1 (*URA3)* | [44] |

44. Schmidt A, Beck T, Koller A, Kunz J, Hall MN. The TOR nutrient signalling pathway phosphorylates NPR1 and inhibits turnover of the tryptophan permease. EMBO J. 1998;17: 6924–31. doi:10.1093/emboj/17.23.6924

73. Sikorski RS, Hieter P. A system of shuttle vectors and yeast host strains designed for efficient manipulation of DNA in Saccharomyces cerevisiae. Genetics. 1989;122: 19–27.
